# Characterization of a Novel Species of *Legionella* Isolated from a Healthcare Facility: *Legionella resiliens* sp. nov

**DOI:** 10.3390/pathogens13030250

**Published:** 2024-03-14

**Authors:** Sandra Cristino, Maria Rosaria Pascale, Federica Marino, Carlo Derelitto, Silvano Salaris, Massimiliano Orsini, Stefano Squarzoni, Antonella Grottola, Luna Girolamini

**Affiliations:** 1 Department of Biological, Geological, and Environmental Sciences, University of Bologna, Via San Giacomo 12, 40126 Bologna, Italy; mariarosaria.pascal2@unibo.it (M.R.P.); federica.marino20@unibo.it (F.M.); carlo.derelitto@unibo.it (C.D.); luna.girolamini2@unibo.it (L.G.); 2 European Society of Clinical Microbiology and Infectious Diseases (ESCMID), Study Group for *Legionella* Infections (ESGLI), 4001 Basel, Switzerland; grottola.antonella@aou.mo.it; 3 Department of Civil, Chemical, Environmental and Materials Engineering, University of Bologna, Viale del Risorgimento 2, 40136 Bologna, Italy; 4 Unit of Biostatistics, Epidemiology and Public Health, Department of Cardiac, Thoracic, Vascular Sciences, and Public Health, University of Padova, Via Loredan, 18, 35121 Padova, Italy; silvano.salaris@ubep.unipd.it; 5 Laboratory of Microbial Ecology and Genomics of Microorganisms, Istituto Zooprofilattico Sperimentale delle Venezie, 35020 Legnaro, Italy; 6 Unit of Bologna, CNR Institute of Molecular Genetics “Luigi Luca Cavalli-Sforza”, 40136 Bologna, Italy; squarzoni@area.bo.cnr.it; 7 IRCCS Istituto Ortopedico Rizzoli, 40136 Bologna, Italy; 8 Regional Reference Laboratory for Clinical Diagnosis of Legionellosis, Unit of Molecular Virology and Microbiology, Modena University Hospital, 41124 Modena, Italy

**Keywords:** *Legionella resiliens* sp. nov., new species, whole genome sequencing (WGS), aquatic environment, polyphasic taxonomy

## Abstract

Two *Legionella*-like isolates, 8cVS16^T^ and 9fVS26, were isolated from a water distribution system (WDS) in a healthcare facility. Cells were Gram- and Ziehl Neelsen-stain-negative, rod-shaped, motile, and exhibited a blue-white fluorescence under Wood’s lamp at 365 nm. The strains grew in a range of 32–37 °C on BCYE with *L-cysteine* (C*ys*+), GVPC, and MWY agar medium, with a positive reaction for oxidase, catalase, and gelatinase. The dominant fatty acids were summed features 3 (C_16:1_ω7c/C_16:1_ω6c) (27.7%), C_16:0_ iso (17.5%), and C_16:0_ (16.3%), and Q13 as the major ubiquinone. The *mip* and *rpoB* gene sequences showed a similarity of 96.7% and 92.4%, with *L. anisa* (ATCC 35292^T^). The whole genomes sequencing (WGS) performed displayed a GC content of 38.21 mol% for both. The digital DNA-DNA hybridization (dDDH) analysis demonstrated the separation of the two strains from the phylogenetically most related *L. anisa (*ATCC 35292^T^), with ≤43% DNA-DNA relatedness. The Average Nucleotide Identity (ANI) between the two strains and *L. anisa* (ATCC 35292^T^) was 90.74%, confirming that the two isolates represent a novel species of the genus *Legionella*. The name proposed for this species is *Legionella resiliens* sp. nov., with 8cVS16^T^ (=DSM 114356^T^ = CCUG 76627^T^) as the type strain.

## 1. Introduction

*Legionella* are Gram-negative aerobic bacilli belonging to the *Legionellaceae* family. The family Legionellaceae is located within the gamma subdivision in the order of Legionellales. The genus *Legionella* was defined for the first time in 1979, and consisted of a single species reported as *Legionella pneumophila* (*Lp*). Since then, of the “72 child taxa” reported in the List of Prokaryotic names with Standing in Nomenclature (LPSN)-approved list of bacteria names, 65 species of *Legionella* have been validly recognized, while others show unvalidated names [1,2,3]. Among the validated species, *L. anisa*, *L. bozemanii*, *L. cherrii*, *L. dumoffii*, *L. gormanii*, *L. lytica*, *L. parisiensis*, *L. rowbothamii*, *L. steigerwaltii*, and *L. tucsonensis* exhibit blue-white autofluorescence under long-wave UV light, while *L. erythra*, *L. rubrilucens*, and *L. taurinensis* show a dark-red autofluorescence. On the other hand, *Lp*, the representative species of the genus *Legionella*, does not exhibit autofluorescence. The auto-fluorescence characteristic is useful for discrimination and identification among *Legionella* species. Therefore, several studies have proposed that *L. bozemanii*, *L. dumoffii*, and *L. gormanii* belong to the *Fluoribacter* genus [4,5].

Classification at the genus level has been controversial; most authors reported a single genus, *Legionella*, but others have proposed division into three genera: *Legionella*, *Tatlockia*, and *Fluoribacter*. The phylogenetic studies based on 16s rRNA, *mip*, and *rpoB* genes and fatty acid profiles confirm that the genera *Legionella* and *Fluoribacter* should be a single monophyletic group, in which most blue-white fluorescent organisms are positioned in a single cluster [1,5,6,7,8,9].

*Legionella* lives in fresh water and is frequently found in free-living form or within biofilms in artificial water distribution systems, considered the main source of *Legionella* infections [1]. From a natural or artificial environment, it can spread via aerosol, infecting human beings. The inhalation of the contaminated aerosol may result in Legionnaires’ disease (LD) or Pontiac Fever [1,10,11]. Considering that all *Legionellae* are able to grow intracellularly in host cells, it is assumed that several species can cause disease in humans, when some conditions occur: immunocompromised patients, smokers, elderly people, etc. [1,12].

Currently, 24 of the 65 species of *Legionella* are associated with disease, with the majority of the cases related to the *Lp* serogroup 1 (*Lp*1) [3,13]. The last ECDC Legionnaires’ disease Annual Epidemiological Report showed a higher incidence of cases in 2021, with 2.4 cases per 100,000 population [14]. Among 1133 (11%) culture-confirmed cases with the pathogen reported, a total of 32 cases (3%) were associated with *L. anisa*, *L. bozemanii*, *L. longbeachae*, *L. micdadei*, and *L. cincinnatiensis*, and 14 were reported as *Legionella* species unknown. The underestimation of *Legionella* cases remains linked to the low culture technique on clinical specimens and the few diagnostic tools developed for *Legionella* non-*pneumophila* species.

Moreover, the epidemiological and phylogenetic data suggest that some cases occurred in northwest Europe and are associated with *Lp* emergent sequence types (ST), such as ST 1, 23, 37, 47, and 62, developed from different genomic backgrounds [15]. Simultaneously, some newly reported cases were associated with new species developed in the natural environment, which promote several mechanisms for spreading in a man-made environment [16,17]. Therefore, environmental surveillance has the aim of detecting these environmental niches and the mechanisms for species evolution. Moreover, the number of species and serogroups of Legionellae continues to increase, especially after the era of next generation sequencing (NGS) technologies, mainly with the introduction of one of the most popular NGS applications: whole genome sequencing (WGS). Regarding these new species, there is little information, especially on their pathogenicity and response to antibiotic treatment based on azithromycin or levofloxacin used in the clinical routine [18]. Moreover, this aspect is relevant considering the absence of standardized antimicrobial susceptibility testing (AST) for *Legionella* treatment, and to date the studies have focused mainly on *Lp*. For these reasons, it is necessary to identify the best methods for detecting *Legionella*, especially the new species, in water systems and in clinical specimens, in order to improve prevention strategies, clinical diagnosis, and also antibiotic treatments. Additionally, an in-depth investigation of *Legionella* distribution in the hospital as well as in the community environment is required in light of the selective pressure performed by environmental climate change other than disinfection treatments often occurred [19]. WGS has become an important tool for determining the source of *Legionella* infections and to understand routes of transmission and selection mechanisms for new pathogenic clones and species.

This study presents a taxonomy characterization of two strains isolated from hot-water distribution systems of a healthcare facility (8cVS16^T^ and 9fVS26) during routine *Legionella* environmental surveillance. The methods to characterize the new species are based on traditional culture, phenotypical and biochemical techniques, and also on the most recent WGS application.

Here, the strains belonging to the genus *Legionella* are presented as novel species under the proposed name of *Legionella resiliens* sp. nov., with strain 8cVS16^T^ proposed as the type strain (=DSM 114356^T^ = CCUG 76627^T^).

## 2. Materials and Methods

### 2.1. Isolation of Bacterial Strains and Growth Conditions

Water samples were collected during an environmental surveillance program, developed from 2012, in a healthcare facility in Bologna, Emilia-Romagna region, Italy, following Italian and Regional Guidelines [20,21,22]. The geographical coordinates are latitude 44°30′2.92″ N, 44°48′12″ E. The healthcare facility’s water distribution system (WDS) showed cold and hot water temperatures of 21.3 ± 0.48 °C and 44.43 ± 0.38 °C, respectively. Moreover, the hot water circuit was treated with hydrogen peroxide and silver salt, with a mean concentration at outlets of 20 mg/L. Briefly, two liters of hot and cold water samples were collected in a post-flushing modality according to the UNI EN ISO 19458:2006 [23]. Following the ISO 11731:2017, *Legionella* isolation was performed using a standard culture technique on Glycine, Vancomycin, Polymyxin B, Cycloheximide (GVPC) agar medium (Thermo Fisher Scientific, Diagnostic, Ltd., Basingstoke, UK) [24]. The plates were incubated at 35.5 ± 2 °C with 2.5% CO_2_ for 15 days and they were observed every two days to control *Legionella* growth.

The two strains were isolated during two different sampling campaign performed in 2015 (9 March 2015) and 2016 (7 March 2016), in hot water samples collected in two different in-patient rooms, both located on the ground floor of the building.

The strain 8cVS16^T^ was found in 2015, in the shower of the bathroom, at a concentration of 200 cfu/L. The temperature of the hot water was 45.8 °C and the disinfectant residue was 20 mg/L. Successively, in 2016, in another in-patient room, a second strain, 9fVS26, was isolated from the toilet shower of the bathroom at a concentration of 250 cfu/L. The temperature of the water sample was 44.0 °C and the disinfectant residue was 20 mg/L.

The putative *Legionella* colonies were then sub-cultured on Buffered Charcoal Yeast Extract (BCYE) agar with L-cysteine (Cys+) and without L-cysteine (Cys−).

The growth conditions at different temperatures, 32, 35.5, and 37 °C, were evaluated on BCYE Cys+, with and without 2.5% of CO_2_, other than in microaerophilia conditions. The colonies were also stored at −80 ± 2 °C in glycerol for further analysis [24]. The morphology of the colonies was studied with a Heerbrugg Wild M38 Professional Optical Stereo Binocular Microscope with Volpi Intralux 4000 Light Source (90 W).

### 2.2. Identification by Serological Test and MALDI–TOF MS Technique

The *Legionella* colonies grown only on BCYE Cys+ plates were identified using the *Legionella* latex agglutination test kit (Thermo Fisher Scientific, Ltd., Basingstoke, UK), according to the manufacturer’s instructions. This test discriminates between *Lp*1, *Lp* serogroups 2–14 (*Lp*2-14), and seven *Legionella* non-*pneumophila* species (n-*Lp*).

Moreover, the colonies were also identified using a Matrix-Assisted Laser Desorption/Ionization–Time of Flight (MALDI) Biotyper system^®^ (Bruker Daltonik GmbH, Bremen, Germany), as previously described [25]. Spectra acquisition and processing were performed using the Microflex LT mass spectrometer (2000–20,000 Da, linear positive mode) and the MALDI Biotyper Compass 4.1 software, whose library (version BDAL revision K (2022)) included the spectra of 48 *Legionella* strains. Data were interpreted according to the manufacturer’s instruction: high confidence level (log score ≥ 2.0), low confidence level (log score between 1.7 and 1.99), not identified (log score between 0.00 and 1.69). A dendrogram based on a Hierarchical Cluster Analysis (HCA) of the MALDI Biotyper spectra was developed using the MALDI Biotyper Compass Explorer software to generate tree-like structures able to link the *Legionella* strains to each other using a linkage algorithm.

### 2.3. Physiology and Chemotaxonomy

The growth of isolates 8cVS16^T^ and 9fVS26 was performed on Wadowsky Yee Medium (MWY), tryptone soya agar (TSA) with 5% sheep blood agar medium (Thermo Fisher Scientific, Diagnostic, Ltd., Basingstoke, UK), and Chocolate Enriched Agar Medium (MEUS S.r.L., Piove di Sacco, Padova, Italy) to observe their growth rate and morphology. The subculture of 8cVS16T, 9fVS26, and *L. anisa* strain WA-316-C3 ATCC 35292^T^ (*L. anisa* ATCC 35292^T^) as the most related strain and *Legionella pneumophila* sg1 strain Philadelphia-1 ATCC 33152^T^ (*Lp*1 ATCC 33152^T^) as the most virulent strain for the subsequent analysis was performed on BCYE Cys+ agar plates.

Gram and Ziehl Neelsen staining were performed on the strains, while the presence of autofluorescence was assessed under Wood’s lamp (long-wavelength UV light at 365 nm).

Additionally, the following biochemical patterns were tested using an oxidase test strips (Biolife, Milan, Italy) and catalase Colorimetric Activity Kit (Thermo Fisher Scientific Diagnostic, Ltd., Basingstoke, UK), which were carried out to analyze the oxidase and catalase activity, respectively. The Diatabs kit and Nutrient Gelatin medium were utilized to evaluate hippurate and gelatinase reactions, respectively (Biolife, Milan, Italy). Moreover, the biochemical strain reactions were investigated using a BBL Crystal Enteric/Non-Fermenter ID kit (Becton Dickinson Systems, Cockeysville, MD, USA) and Remel RapID NF Plus system (Thermo Fisher Diagnostic) following the manufacturer’s protocol. The identification of β-lactamase production was assessed by Oxoid™ Nitrocefin Solution (Thermo Fisher Scientific).

Scanning Electron Microscopy (SEM) was used to describe the ultrastructural morphology of the cell of the strain. Briefly, a loop containing the strain grown on BCYE Cys+, as well as a fragment of culture medium (5 × 5 mm), directly cut from the plate, were transferred onto coverslips. Subsequently, both were directly fixed with glutaraldehyde 2.5% in cacodylate buffer at 0.1 M pH 7.4 for 48 h at room temperature, rinsed with Cacodylate buffer 0.1 M pH 7.4, and post-fixed with OsO_4_ 1% in cacodylate buffer 0.1 M pH 7.4 for 1 h at 4 °C. The sections were then dehydrated in an ethanol series at room temperature and dried by critical-point-drying in a Balzers CPD 030 apparatus. The samples were then mounted on aluminum stubs with silver adhesive paint, sputtered with gold in an Edwards S150B apparatus and observed with a Zeiss EVO MA10 SEM (Oberkochen, Germany) at 20 kV.

In addition, analyses of the composition of cell wall fatty acids (CFAs), isoprenoid quinones, polyamines, and lipids of 8cVS16^T^, 9fVS26, and *L. anisa* strain FDAARGOS DSM 17627^T^ (*L. anisa* DSM 17627^T^) were carried out by Identification Services, Leibniz-Institut DSMZ—Deutsche Sammulung von Mikroorganismen und Zellkulturen GmbH, Braunschweig, Germany. The polyamine profile was obtained by an extraction process from 50 to 60 mg of wet biomass and analyzed via gas chromatography–mass spectrometry (GC-MS). The polyamines and precursors screened included agmatine, cadaverine, homospermidine, norspermidine, 1,2- and 1,3-diaminopropane, putrescine, N-acetyl-putrescine, spermidine, and spermine. The data of the most related *Legionella* strains and *Lp* subs. *pneumophila* Philadelphia 1 as positive control (CCUG 9568^T^) were obtained from the literature [26,27,28].

### 2.4. DNA Extraction and Gene Sequencing

Two colonies, randomly chosen from the samples with a positive result in 2015 and 2016, named 8cVS16^T^ and 9fVS26, were processed using *mip*, *rpoB*, and 16S rRNA gene sequencing [6,7,29]. DNA extraction was performed using InstaGene Matrix (Bio-Rad, Hercules, CA, USA) and quantified with a Qubit fluorometer (Thermo Fisher Scientific, Paisley, UK). The gold standard for the identification of *Legionella* spp., in clinical and in environmental samples, is represented by the sequencing of the macrophage infectivity potentiator (*mip*) gene [22]. The *Mip* gene encodes for a 24 kDa surface protein serving as an essential virulence factor during the invasion process of *Legionella* in the host cells [30]. The protocol used for *mip* gene sequencing was provided by the European Society of Clinical Microbiology and Infectious Diseases (ESCMID) Study Group for *Legionella* Infections (ESGLI) [7,31].

Moreover, RNA polymerase beta subunit (*rpoB*) gene sequencing was used for the identification of isolates, considering the higher discriminant power with respect to the *mip* gene [32]. This gene encodes for a subunit of DNA-dependent RNA polymerase that includes a highly conserved region throughout the bacteria that may be used for bacterial classification [33]. The protocols for *mip* and *rpoB* genes’ PCR amplification were carried out as previously described [6,7,21].

Despite the fact that the use of the 16S rRNA gene for *Legionella* identification was surpassed, it was analyzed following the protocol described by Rafiee et al. considering the requirements for novel species description [34].

Following purification with an ExoSAP-ITTM PCR Product Cleanup kit (Applied Biosystems, Foster City, CA, USA), *mip*, *rpoB*, and 16S rRNA amplicons were sequenced using BigDye Chemistry and analyzed on an ABI PRISM 3100 Genetic Analyzer (Applied Biosystems, Foster City, CA, USA). Raw sequencing data were assembled using CLC Main Workbench 22.0.2 software (QIAgen, Hilden, Germany).

Regarding the gene sequences’ similarities among our two strains and other *Legionella* species, officially recognized [3] and available in the culture collections, Basic Local Alignment Search Tool (BLAST) (http://blast.ncbi.nlm.nih.gov/Blast.cgi, accessed on 22 July 2020) research from the National Center for Biotechnology Information (NCBI) was carried out to obtain the best match for *Legionella* identification using the PCR amplicons of *mip* (611 bp), *rpoB* (329 bp), and 16S rRNA (1468 bp) genes.

The *mip* sequences were also compared to the deposited sequences in the *Legionella mip*-gene sequence database, using a similarity analysis tool. ESGLI has established an accessible web database (http://bioinformatics.phe.org.uk/cgi-bin/Legionella/mip/mip_id.cgi, accessed on 22 July 2020) that contains sequence data from described species and allows the identification of *Legionella* species (this link is undergoing development and is currently unavailable externally but can be accessed internally by the database curators at UKHSA (legionella-sbt@ukhsa.gov.uk)). Considering the classification scheme targeting the *mip* gene sequence developed by Ratcliff et al., species-level identification was performed based on a similarity score of >98.0% [7,31].

Regarding *rpoB* gene, the sequences were compared to the type strain sequences deposited in NCBI from several culture collections, including the American Type Culture Collection (ATCC), the National Collection of Type Cultures, the Central Public Health Laboratory (NCTC), the NITE Biological Research Center, the National Institute of Technology and Evaluation (NBRC), Deutsche Sammlung von Mikroorganismen und Zellkulturen (DSM), etc. In relation to the new *Legionella* classification scheme targeting the *rpoB* gene, developed on a gene fragment of 329 bp, proposed by Pascale et al., the species-level identification was performed based on the basis of a similarity score fixed at >95.2% [32].

Concerning the 16S rRNA gene sequence similarity, the threshold percentage of species-level identification was set at >97% [35], although more recently a study by Stackebrandt and Ebers set a more relaxed cut-off value, set at >98.7 [36].

Moreover, the gene sequences’ similarity was also assessed using the entire gene sequences obtained by the WGS results.

### 2.5. Phylogenetic Analyses Based on Gene Sequences

A multiple sequence alignment (MSA) and a phylogenetic tree for *mip*, *rpoB*, and 16s rRNA genes sequences were built to estimate the relationship among 8cVS16^T^ and 9fVS26 strains. When necessary, manual editing was conducted on the sequences, trimming them to the same length as the reference sequence. In addition, BLAST searches on NCBI were carried out to obtain the top ten strain identification results.

The nucleotide sequences were aligned by a multiple sequence comparison using the log-expectation (MUSCLE) algorithm [37], performed in Geneious Prime genome browser implemented with 2023.0.4 software (http://www.geneious.com, accessed on 6 July 2023) [38], retaining the default settings. The phylogenetic trees were built with the aligned sequences that were passed on to Bayesian Evolutionary Analysis by Sampling Trees (BEAST) (v. 1.10.4) [39]. The consensus trees were chosen by Bayesian Evolutionary Analysis Utility (BEAUti) (v. 1.10.4) [40].

### 2.6. Whole Genome Sequencing and Genome Features

WGS of the two strains (8cVS16^T^ and 9fVS26) was carried out as previously described [21]. Briefly, NGS library preparation was performed with 100 ng of genomic DNA, using the Nextera XT DNA library prep kit (Illumina, New England Biolabs, Ipswich, MA, USA). The sequencing was performed using the Illumina NextSeq 500 platform (2 × 250 paired-end reads). Sequencing reads were processed by the TORMES (v.1.2.0) pipeline set at default parameters [41], to obtain an assembly at the level of the draft genome. The pipeline carried out sequence quality filtering (PRINSEQ v. 0.20.4) and a de novo genome assembly (SPAdes v. 13.4.1) [42].

The assembly generated by Tormes was scaffolded using CSAR (v1.1.1) [43], to improve the draft genome quality, using a reference-based approach. *Legionella* sp. PC1000 (NZ_CP059400.1) was chosen as a reference organism. Geneious Prime 2022.0.2 software (http://www.geneious.com, accessed on 6 July 2023) was used to perform further refinement, remapping the reads obtained by CSAR scaffolds. The evaluation of the completeness of the two genome assemblies was performed by Benchmarking Universal Single-Copy Orthologs (BUSCO) (version 5.0.0) [44]. The final draft genomes were submitted to the GenBank, requiring the annotation by the PGAP pipeline (v4.3) [45]. Moreover, the completeness and contamination of the two genomes were assessed by CheckM (v1.1.6) [46].

Genomic similarities among the assembled draft genomes were calculated using the OrthoANI package [47]. Furthermore, the average nucleotide identity (ANI) value was also measured using FastANI [48] through DFAST [49], comparing our strains against 13,000 prokaryotic reference genomes from NCBI. Furthermore, ANIb and ANIm through JSpeciesWS (v. 3.9.8) (https://jspecies.ribohost.com/jspeciesws/#home, accessed on 8 November 2021) [50], using, respectively, BLAST+ and MUMmer as a comparative algorithm, were calculated. The Genome-to-Genome Distance Calculator 3.0 (GGDC) web service (https://ggdc.dsmz.de/ggdc.php#, accessed on 8 November 2021) [51] was used to analyze the phylogenetic relationships, applying a DNA-DNA hybridization (dDDH) analysis. Parameters were kept as default values. The GGDC findings were based on formula 2, which is suitable for use with incomplete draft genomes and is independent of genome length. BLAST+ was used as a local alignment tool [52]. A comparison was made between our strains and *L. anisa* (ATCC 35292^T^), the closest related strain based on the previous ANI outcomes. A relationship between our strains and the other 63 *Legionella* species’ WGSs annotated in NCBI was assessed by the Codon Tree pipeline using Phylogenetic Tree Service—Bacterial and Viral Bioinformatics Resource Center (BV-BRC) (v3.30.19, https://www.bv-brc.org/app/PhylogeneticTree, accessed on 6 July 2023) [53]. The list of genome sequences used is shown in Table S1, present in the supplementary information. MUSCLE [37] was used to align the protein sequences, while the Codon align function of BioPython was used to align the nucleotide coding gene sequences [54]. A concatenated alignment, provided by MAFFT, of all proteins and nucleotides was passed to RaxML (v. 8.2.11) [55]. Support values were generated using 100 rounds of the “Rapid” bootstrapping option of RaxML [56]. The resulting file was examined in FigTree (v1.4.4) (http://tree.bio.ed.ac.uk/software/figtree/, accessed on 6 July 2023) to generate a good-quality image and the tree was modelled and rooted by midpoint-rooting.

The isolates’ clonality was assessed using RAPD-PCR and BOX-PCR fingerprinting. The undiluted and 70 ng of DNA template were amplified using REP1R-Dt (3′-CGGNCTACNGCNGCNIII-5′) and REP2-Dt (3′-CATCCGGNCTATTCNGCN-5′) primers, according to GEORGHIOU et al. [57], and primer BOXAR1 (59-CTACGGCAAGGCGACGCTGACG-39) according to Michelini et al. [58]. The amplicons were separated by electrophoresis in 2% (*w*/*v*) agarose gel. The strain of *B. longum* subsp. *longum* B 1478 was used as a positive control. Moreover, the differences in number of SNPs between the two strains were calculated using Snippy v.4.6.0.

### 2.7. Core Genome

The main genomic data of the most related *Legionella* species and the most virulent *Lp*1, provided by NCBI, were compared with 8cVS16^T^ and 9fVS26 strains.

Additionally, an in-depth analysis of the two strains was provided by BLAST Ring Image alikhan 2011Generator (BRIG) (v. 0.95) software, to compare their genomes with *L. anisa* (ATCC 35292^T^) and *Legionella pneumophila* subs. *pneumophila Philadelphia serogroup 1* (*Lp1*) (*ATCC33152^T^*) (*Lp1* ATCC 33152^T^), using *L. anisa* as the reference genome [59]. Moreover, the analysis of differences in the genome size and sequences were investigated by Prokka annotation pipeline software (v. 1.14.6). The missing genes were analyzed among the four genomes using in-house Python script.

The pangenome analysis contributes to the construction of an overview of genes that are shared among all the species of *Legionella* and those that are present in only a few genomes [60]. Therefore, a pangenome analysis was performed by Roary software (v. 3.13.0) [61]. The genes were classified as follows:

- Total genes (0% ≤ strains ≤ 100%);
- Core genes (99% ≤ strains ≤ 100%);
- Soft core genes 95% ≤ strains < 99%);
- Shell genes (15% ≤ strains < 95%);
- Cloud genes (0% ≤ strains < 15%).

A comparative amino acid analysis was performed among our strains and the other 63 *Legionella* species genome through Average Amino acid Identity (AAI) performed by EzAAI tool (v. 1.2.1 http://leb.snu.ac.kr/ezaai/download, accessed on 7 July 2023) to determine the overall similarity among the genomes [62]. Successively, the AAI data were represented in a heatmap built by Python script (v. 3.10.4) using Matplotlib (v. 3.6.3), Pandas (v. 1.4.2), and Seaborn (v. 0.12.2) libraries.

In order to study the number of single-copy orthologous protein sequences in common among the 63 *Legionella* species, a Venn diagram was built using different clustering algorithms such as Bi-Directional Best-Hits (BDBH) [63], COGtriangle (COG) [64], and OrthoMCL (OMCL) [65] by Get_homologue [63].

### 2.8. Plasmid, Virulence, Pathogenicity, and Antibiotic Resistance Gene Analysis

The chromosome assembly of the most related genome (*Legionella* sp. PC1000: NZ_CP059400.1) to our strain included three plasmids: pPC1000_1: CP059577, pPC1000_2: CP059401, and pPC1000_3: CP059402. Therefore, their sequences were retrieved from PLSDB (v. 2021_06_23_v2) [66], a plasmid database, and used as a reference to map our reads through Geneious software. The analysis of virulence and antibiotic resistance gene tracts were annotated by TORMES pipelines and Rapid Annotation using the Subsystem Technology (RAST) (v2.0) server [67]. In particular, the ABRicate (v. 1.0.0) tool (https://github.com/tseemann/abricate, accessed on 6 July 2023) was utilized by TORMES to find virulence genes by screening the genome against the Virulence Factors Data Base (VFDB) (http://www.mgc.ac.cn/VFs/main.htm, accessed on 6 July 2023) [68]. Furthermore, ABRicate was also used to screen the antibiotic resistance genes against three databases: ARG-ANNOT (v.28 July 2019) [69], CARD (v.2.1.2) [70], and ResFinder 3.2.0 [71]. In conclusion, the potential pathogenicity of two strains was putatively determined using PathogenFinder (v 1.1) [72]. Except as otherwise specified, every software was run with its default settings.

### 2.9. Antibiotic Susceptibility Test

Regardless of the absence of a technical guideline to determine the minimum inhibitory concentrations (MICs) and the ‘epidemiological cut-off’ (ECOFF) values for *Legionella*, the MIC Test Strip and Broth Microdilution (BMD) techniques were performed on 8cVS16^T^ and 9fVS26 strains to assess their antibiotic susceptibility pathways.

The MIC Test Strip (Liofilchem, s.r.l, Roseto degli Abbruzzi (TE), Italy) was performed on subcultures of the strains grown on BCYE Cys + agar at 35 °C with 2.5% of CO_2_ in a humidified atmosphere for 48 h. The strains were then resuspended in sterile water to obtain a 0.5 McFarland turbidity solution. Consecutively, the suspensions were plated on the entire surface of the BCYE Cys+ plate with a swab and a single gradient strip was applied to the medium. This step was iterated for each antibiotic tested: azithromycin (0.016–256 mg/L), erythromycin (0.002–32 mg/L), ciprofloxacin (0.016–256 mg/L), rifampicin (0.016–256 mg/L), tigecycline (0.002–32 mg/L), and imipenem (0.002–32 mg/L). The concentration range tested is indicated in parenthesis. The plates were then incubated for 48 h at 35 °C in a humidified atmosphere. The MICs were read at the point of intersection between the growth of the biomass of the colony and the gradient strip. The interpretation of MICs was carried out by comparing the MIC obtained for the isolate tested with the MIC reference table provided by EUCAST (European Committee on Antimicrobial Susceptibility Testing) guidance documents, produced by ESCMID [73].

Regarding the BMD Method, the test was performed using a suspension of the strains in liquid growth medium (LGM), without charcoal to avoid the inactivation of antibiotics [74], producing a 0.5 McFarland turbidity solution. In 96-well microtiter plates, 40 µL of antibiotic solution with 160 µL of bacterial suspension were plated and each line of the microtiter plates contained one antibiotic. The antibiotics tested were the following: azithromycin (0.0075–16 mg/L), erythromycin (0.0075–16 mg/L), and ciprofloxacin (0.00025–0.512 mg/L). The concentration range tested is indicated in parenthesis.

The final bacterial concentration was 4 × 10^5^–5 × 10^5^ cells/mL. Successively, the plates were incubated at 35 °C for 48 h in a humidified atmosphere. The interpretation of MICs was carried out by comparing the MIC obtained for the isolate with the MIC distribution table provided by EUCAST guidance documents [73].

## 3. Results and Discussion

During the study, both 8cVS16^T^ and 9fVS26 strains were used to test their morphological, biochemical, and genotypical characteristics. Moreover, *L. anisa* (ATCC 35292^T^) and *Lp*1 (ATCC33152^T^) were used as the most related and virulent strains, respectively. The main features of the most related strains clade were also taken from the reference literature.

### 3.1. Isolation of Bacterial Strains, Growth Conditions, and Identification

**Figure 1:**
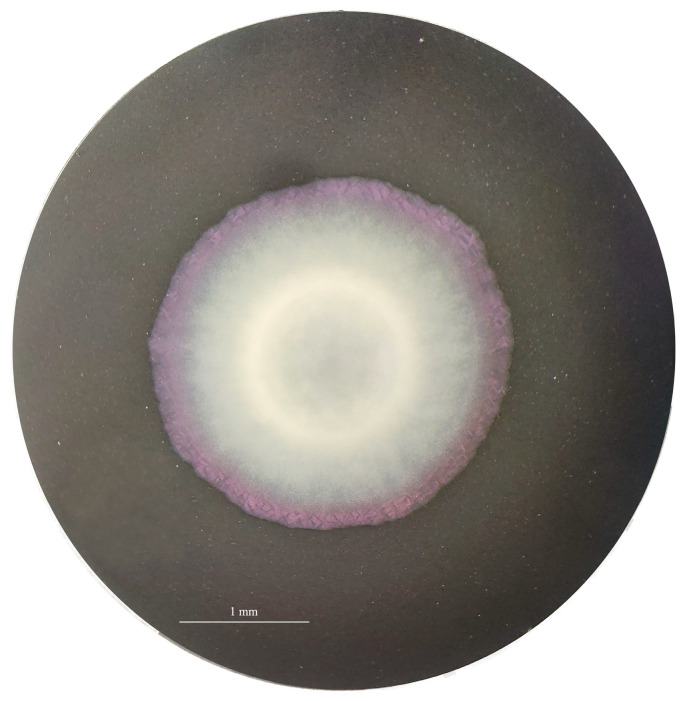
Single colony of 8cVS16^T^ growth on BCYE Cys+ medium for 48 h at 35 °C. Image acquired using a Heerbrugg Wild M38 Professional Optical Stereo Binocular Microscope with Volpi Intralux 4000 Light Source (90 W). Magnification ×10 and continuous zoom magnification ×4.5.

**Figure 2:**
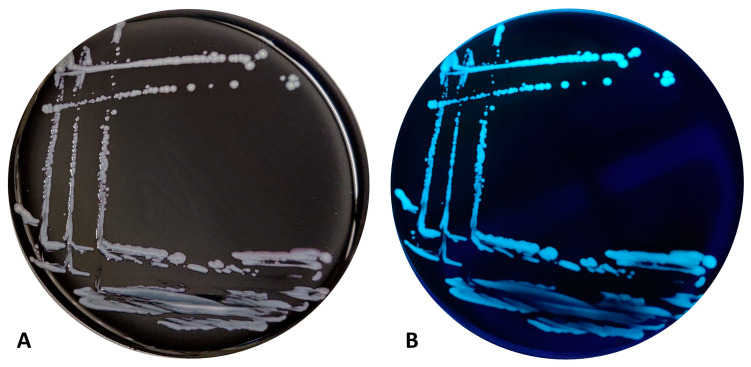
Strain 8cVS16^T^ growth on BCYE Cys+ for 48 h at 35 °C and 2.5% CO_2_ (**A**) and under Wood’s lamp (long-wavelength UV light at 365 nm) (**B**).

The *Legionella*-like colonies showed growth only on GVPC, BCYE C*ys*+, and MWY. No growth was observed on other media (Figure S1). Moreover, the best growth was observed at 32 and 35.5 °C, compared to 37 °C, without differences between the presence of CO_2_, with respect to microaerophilia. The sub-culture of the strains on BCYE Cys+ medium displayed a rapid growth at 35.5 ± 2 °C with 2.5% CO_2_ after 24 h of incubation. The colonies were convex and light grey with a pinkish contour and round shape, with an approximate diameter of 2 mm (Figure 1 and Figure 2).

The strains showed blue-white autofluorescence under a Wood’s lamp (long-wavelength UV light at 365 nm). The fluorescence was lost during growth at 37 °C for 24 h on BCYE Cys+ medium, as well as after the defrost process.

The colonies showed a positive result for *Legionella* species antisera using the *Legionella* latex agglutination test (*Legionella* latex test kit; Thermo Fisher Scientific, Ltd., Basingstoke, UK).

**Figure 3:**
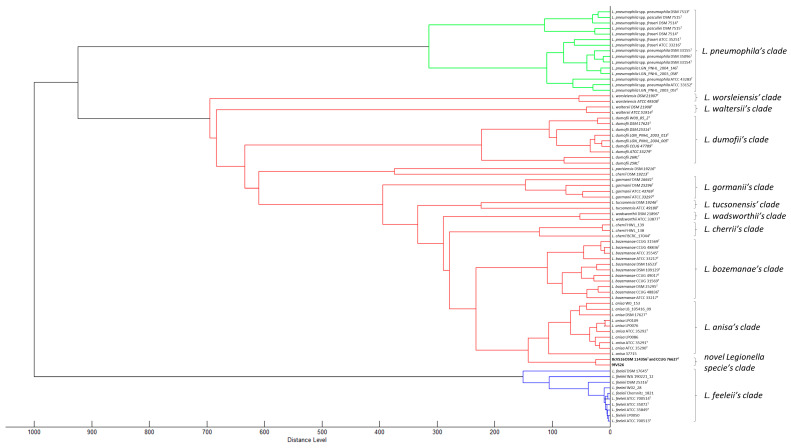
Dendrogram based on whole-cell MALDI-TOF mass spectra (Maldi Byotyper, Bruker^®^) of strains 8cVS16^T^ and 9fVS26 (in bold type) and other *Legionella* strains present in the instrument data base.

The MALDI Biotyper System^®^ identified both strains as *L. anisa*, with a low confidence score, returning values of 1.76 and 1.78 for 8cVS16^T^ and 9fVS26, respectively. The dendrograms elaborated by MALDI-TOF MS (Figure 3 and Figure S2) showed a clear separation of the two strains with respect to the closely related *Legionella* strains (*L. anisa*) and other *Legionella* reference strains, available in the instrument database. In particular, the dendrograms displayed a monophyletic group including *L. anisa* and *L. bozemanae*, where our strains made a separate clade inside the branch.

### 3.2. Physiological, Biochemical, and Morphological Features

The cells of 8cVS16^T^ and 9fVS26 strains were Gram- and Ziehl Neelsen-stain-negative. Light microscopy observation showed the motility of the strains. The strains were positive for oxidase, catalase, and for gelatinase tests. On the other hand, the hippurate test showed a negative response, while β-lactamase production was observed. Table 1 shows the results of the main biochemical parameters tested and their comparison with the closed *Legionella* species and *Lp*1 data [28,75,76,77,78].

The results of the biochemical parameters tested on the two strains *L. anisa* (ATCC 35292^T^) and *Lp*1 (ATCC 33152^T^) are summarized in Table 2.

Additionally, the analysis of CFA composition resulted in a high presence of Summed Features 3 (C_16:1_ ω7c/C_16:1_ ω6c) as a predominant CFA (27.7%), followed by methyl-branched fatty acids C_16:0_ iso (17.5%) and saturated fatty acids C_16:0_ (16.3%). The CFA composition of 8cVS16^T^ and 9fVS26 strains, *Legionella* most-related species, and *Lp*1 are shown in Table S2. Our strains showed a similar profile pattern to that of *L. anisa* (DSM 17627^T^).

Furthermore, regarding the analysis of isoprenoid quinones, the outcomes revealed that the major ubiquinone was Q13 (50.2%) (Table S3). In this case, no similarity with other strains was found. Moreover, the major lipids found were diphosphatidylglycerol (DPG), phosphatidylethanolamine (PE), phosphatidylcholine (PC), and phosphatidylglycerol (PG) for both the 8cVS16^T^ and 9fVS26 strains, and *L. anisa* (DSM17627^T^). A minor amount of unidentified aminolipids (AL), aminophospholipids (APL), and lipids (L) (strain dependent) were also found (Figure S3). Finally, putrescine was the only polyamine detected in our strains and *L. anisa*.

#### Scanning Electron Microscopy (SEM) and Motility Gene Patterns

**Figure 4:**
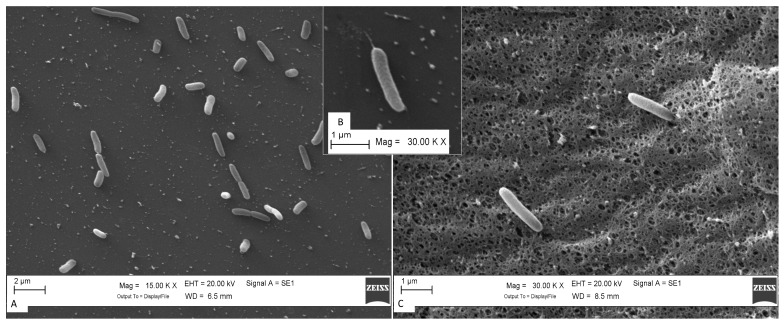
Scanning electron microscopy (SEM) images of strain 8cVS16T grown on BCYE Cys+ agar for 48 h at 35 °C with 2.5% CO_2_. View of (**A**) aflagellate form of strain grown on BCYE Cys+ (Bar 2 μm), (**B**) flagellate form of strain grown on BCYE Cys+, and (**C**) fragment (5 × 5 mm) of BCYE Cys + medium on which the strain grew. Bar (**A**) 2 µm and (**B**,**C**) 1 μm. Magnification: (**A**) ×15,000 and (**B**,**C**) ×30,000.

The cells of strains in SEM images showed a rod shape with an average size of 0.43 ± 0.02 μm wide and 1.45 ± 0.08 μm long, and not all the cells showed flagella. The appearance of cells varied from single cells to chains and groups of two or three (Figure 4A,C). The strains were motile by flagella (Figure 4B).

Regarding the flagella presence, the genome annotation of the sequenced strains showed the presence of genes that regulate the production and motility of flagella in prokaryotes, which is consistent with these findings. The reported genes were *fliM*, *fliQ*, *fliR*, *flhB*, *flhA_1*, *flhF*, *ylxH*, *fliA*, *fliS*, *fliC*, *fliE*, *fliF*, *fliG*, *fliI*, *flgI*, *flgH*, *flgG*, *flgF*, *flgE*, *flgD*, *flgC*, and *flgB.* According to Appelt and Heuner [79], the presence of flagella is strictly related to several environmental factors, including temperature, medium viscosity, and nutrient availability (e.g., fatty acids and amino acids). Moreover, the transitory presence of the flagella could be explained by the presence of *Legionella* cells in the growth phase during the SEM analysis.

### 3.3. Phylogenetic and Sequencing Analyses

The comparison of 16S RNA, *mip*, and *rpoB* sequences obtained by PCR analysis, between the two strains 8cVS16^T^ and 9fVS26, showed an identity percentage and coverage of 100%. The gene sequences’ similarities results were obtained using both PCR and WGS sequences. The data of the top ten results obtained, comparing our two strains and other *Legionella* species officially recognized [3] and available in the culture collections, are displayed in Table 3.

Briefly, the main genomic features reported were:

- Regarding 16SrRNA, using 1537 bp obtained by WGS, the range of similarity with respect to the top ten *Legionella* species was between 97.34 and 99.29%;
- Considering the classification scheme targeting the *mip* sequence for the identification of novel *Legionella* isolates, using 611 bp, the range was 88.93–96.73%;
- In relation to the new classification scheme targeting the *rpoB* sequencing for a deep-resolution identification of the novel *Legionella* isolate, using 329 bp, the range was between 83.07 and 92.40%. In addition, using the entire *rpoB* gene (4107 bp) obtained by WGS, the range of similarity was 80.32–95.13%.

The best match for both strains obtained by BLAST research on NCBI returned *L. anisa* strain (ATCC 35292^T^) (GenBank accession number GCA_900639785.1) with a similarity of 96.7%, with 20 DNA mismatches (mm) and 1 amino acid (AA) mismatch for *mip* (611 bp) and 92.4%, 25 DNA mismatches and 0 AA mismatches for *rpoB* (329 bp). Concerning the entire *rpoB* gene sequence (4107 bp), the result showed an identity of 95.1%, with 200 DNA mm and 13 AA mm with the same *L. anisa* type strain. Starting from the intraspecies identification threshold proposed for the 16sRNA, *mip*, and *rpoB* genes (98.7%, 98.0%, and 95.2%, respectively), the values obtained for the two strains 8cVS16^T^ and 9fVS26 confirmed the genus classification as *Legionella* for the 16sRNA gene. In detail, the match of 99.29% with *L. anisa* DSM 17627T (CP082852.1) demonstrates that the two isolates belong to the *L. anisa* species. Regarding *mip* and *rpoB* gene sequencing, the data found fell within the threshold for intraspecies identification, suggesting that our strains could be considered a novel *Legionella* species.

Moreover, phylogenetic analysis based on the *mip*, *rpoB*, and 16S rRNA gene sequences revealed that 8cVS16^T^ and 9fVS26 formed a clearly separate clade inside the main *L. anisa* clade (Figures S4–S6).

Genome sequences of the type strain 8cVS16^T^ and 9cVS26 were deposited in the GenBank database under the following accession numbers:

- *mip*: MW052957.1 and MW052913.1;
- *rpoB*: MZ367138 and MZ367095;
- 16S rRNAs: OL804581.1 and OL889882.1.

#### Whole Genome Sequencing (WGS) and Comparative Analysis

**Figure 5:**
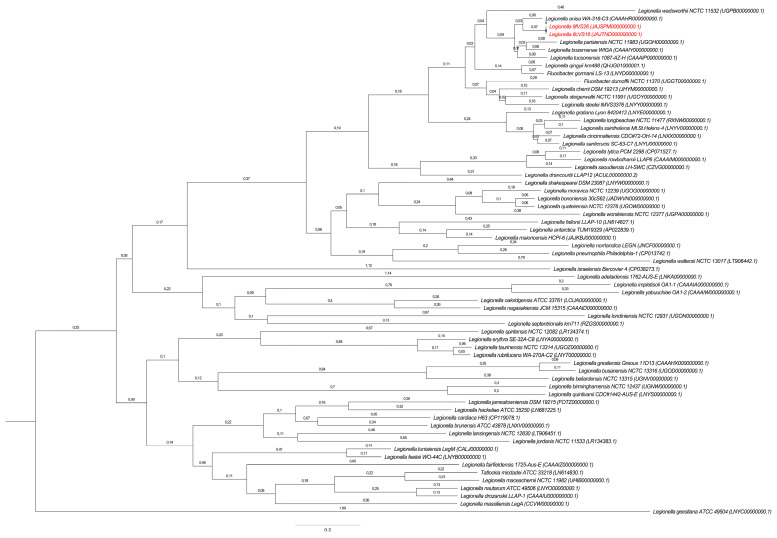
Comparative WGS relationship among 8cVS16^T^ and 9fVS26 and the other 62 *Legionella* species annotated in NCBI. Branch labels display the substitutions per site. The bootstrap values are 100, using the “Rapid” bootstrapping option of RaxML [56]. Bar 0.2 substitution per nucleotide position; 8cVS16^T^ and 9fVS26 are in red.

The same results were obtained using the WGS analysis. In particular, the comparison between 8cVS16^T^ and 9fVS26 strains with *L. anisa* WA-316-C3 (ATCC 35292^T^) confirmed the previous results, reporting ANIb values of 90.08% and 90.09% for 8cVS16^T^ and 9fVS26, respectively, and an ANIm of 91.55% for both strains. The value obtained by the dDDH was 43%, leading to the conclusion that the compared species were distinct from each other, since the in silico DDH threshold for species similarity was above 70% (35). Making an allowance for the thresholds established for ANI (95%) and dDDH (70%) analysis, the results obtained led us to consider the two strains as new *Legionella* species (35,36). The phylogenetic trees obtained by WGS showed a monophyletic group including *L. anisa*, *L. bozemanae*, *L. parisiensis*, and *L. tucsonensis* (Figure 5). However, the tree highlights the presence of a separate clade inside the monophyletic group of *L. anisa* (ATCC 35292^T^) (Figure 5).

Regarding the WGS results, the data regarding assembling and annotation are summarized in Table 4.

Concisely, the total lengths of the genomes were 3,906,083 bp (3.9 Mbp) and 3,906,100 bp (3.9 Mbp) for 8cVS16^T^ and 9fVS26, respectively, with a GC content of approximately 38.2 mol%. The two strains showed a similarity of 99.98%, confirming that the two strains belong to the same species and are identical to each other. Moreover, the completeness and contamination of the two genomes was 98.09% and 0.76% for both strains, respectively. Our values fell within the range suggested by Parks et al., which established a high completeness and low contamination with the following values: ≥90% and ≤5% [46]. The results of the genome comparison between the two strains showed 99.98% identity, proving that the strains are both members of the same species. The assessment of clonality by RAPD-PCR and BOX-PCR confirmed the clonality of the strains (Figure S7). Moreover, the analysis of total genetic diversity (SNP density) produced a difference of 25 SNPs between the two strains, forming a complex of SNPs and multiple dispersed nucleotide polymorphism (MNP), confirming their clonality [80].

In conclusion, the GenBank accession numbers for the whole genome sequencing of the strains 8cVS16^T^ and 9fVS26 were JAJTND000000000 and JAJSPM000000000, respectively.

### 3.4. Core Genome

The main genomic data of the strains 8cVS16^T^ and 9fVS26 compared with the most related *Legionella* species (*n* = 6) are shown in Table 5.

**Figure 6:**
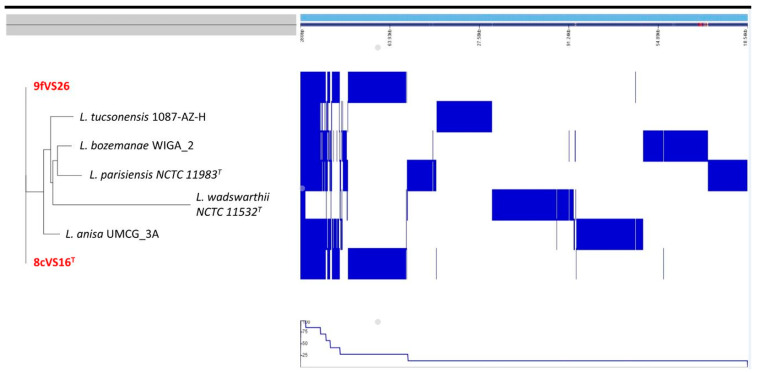
Visualization of the pangenome analysis of the strains 8cVS16^T^ and 9fVS26 obtained with Roary software (v. 3.13.0) and the close phylogenetic relative strains (*L. anisa*, *L. bozemanae*, *L. parisiensis*, and *L. tucsonensis*). Using the presence or absence of core genes, the whole genomes of the strains were clustered. The blue color indicated the presence of the gene, whereas the white color indicated the absence of the gene. The bottom part of the figure represents the genetic frequency of the pangenome.

The information was provided by NCBI. In addition, to better investigate the relationship between our genome strains and *L. anisa* (the most related strain) and *Lp*1 (the most virulent strain), the alignment of their genome was performed, using *L. anisa* as the reference genome. Figure S8 shows that 8cVS16^T^, 9fVS26, and *Lp*1 genomes are shorter than the *L. anisa* one, and contain some missing regions (Table S4). The pangenome results showed that our strains were located in a separate clade than the related and *Lp*1 one (Figure 6).

The classification of the genes for our strains and the most related *Legionella* species was as follows:

- Total genes: 174,828;
- Core genes: 3;
- Soft core genes: 7;
- Shell genes: 484;
- Cloud genes: 174,334.

**Figure 7:**
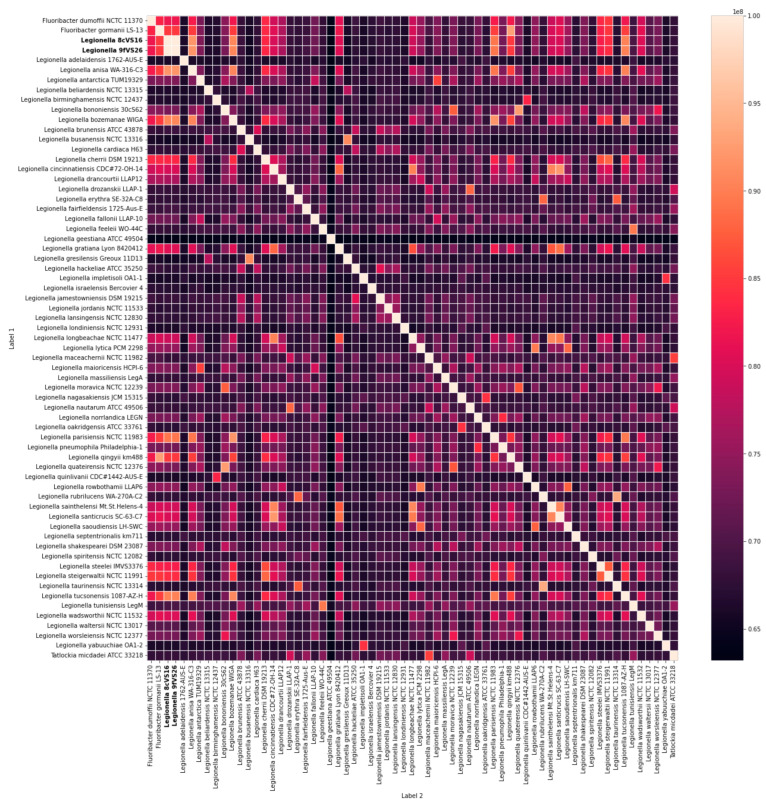
Amino acid composition heatmap (AAI data) of 8cVS16^T^ and 9fVS26 strains (in bold type) and the other 63 *Legionella* species genome presented in NCBI. The heatmap is based on a comparison of amino acid composition, expressed as a percentage, among genomes of *Legionella* species. Table S1 contains a list of the *Legionella* species used. The heatmap colors represent the percentage of similarity, from white (highest value) to dark red (lowest value).

The results obtained by WGS analysis were also confirmed by the heatmap based on the amino acid profile (AAI data). In fact, the amino acid compositions of 8cVS16^T^ and 9fVS26 were close to those of their phylogenetic *Legionella* clade (*L. anisa*, *L. bozemanae*, *L. parisiensis* and *L. tucsonensis*) (Figure 7).

### 3.5. Plasmid, Virulence, Pathogenicity, and Antibiotic Resistance Gene Results

The mapping of our reads on the genomes of the three plasmids of the most related genome (*Legionella* sp. PC1000: NZ_CP059400.1) resulted in a genome completeness of 97.8% with the sequences of only plasmid pPC1000_3. The partial sequence of this plasmid, called pVS16, showed a length of 33,687 bp and a weight of 10,425,647.35 DA. The plasmid was composed of two scaffolds of 23,720 bp and 9967 bp, submitted on NCBI as ON715015.1 and ON715014.1, respectively.

Regarding the presence of virulence genes, the virulence factors reported in our strains included genes for adherence, motility, intracellular survival (*mip*), iron uptake, and regulation. Moreover, genes encoding for stress proteins, invasion, and regulation virulence-related genes (for example, *LetA*/*S* two-component system and *RelA*) were found. A type II Lsp, a type IVA (T4ASS), and IVB *Icm*/*Dot*, together with effector proteins linked to this secretion complex, were also found. The presence of virulence genes was also investigated among the *Legionella* species belonging to the same clade: 8cVS16^T^, 9fVS26, *Legionella anisa* WA-316-C3 ATCC35292^T^, *Legionella bozemanae* WIGA ATCC 33217^T^, *Legionella parisiensis* PF-209C-C2 ATCC35299^T^, *Legionella tusconensis* 1087AZH ATCC 49180^T^, and *Legionella wadsworthii* 81-716A ATCC 33877^T^ (Table S5). All of them showed blue-white autofluorescence.

Regarding the antibiotic resistance genes, the Tormes workflow detected FEZ-1 as a β-lactamase resistance gene. The FEZ-1 gene is also associated with the resistance of carbapenem, cephalosporin, and penam antibiotics. Despite that, they are not conventionally used for legionellosis treatment, and the same resistance has already been reported for *L. gormanii* and *L. anisa* [81,82].

In conclusion, the PathogenFinder study combined with the pathogenetic investigation of the 8cVS16^T^ and 9fVS26 strains for genes and combinations associated with virulence factors identified our strains as potential human pathogens with a probability of 83.7%.

### 3.6. Antibiotic Susceptibility Test Results

The MICs obtained for 8cVS16^T^ and 9fVS26 strains and *Lp*1 (ATCC 33152^T^) with the two methods are shown in Table 6 and Table 7.

Notably, our strains showed a low sensitivity to azithromycin (MIC 1 mg/L) for both methods. This low sensitivity to azithromycin could be explained by the possible implication of efflux pump activity [83] in the phenotype of the strains, which requires further investigation.

## 4. Conclusions

### Description of Legionella resiliens sp. nov.

*Legionella resiliens* (re.si’li.ens. L. part. adj. resiliens, leaping back, resilient). The term “*resiliens*” derives from the Latin “resilire” that indicates the ability to cope positively with traumatic events, to reorganize life positively, and to rebuild oneself without alienating its identity. It represents the spirit that drives all researchers working in the scientific community.

Cells are Gram-stain-negative, Ziehl Neelsen-stain-negative, rod-shaped, and motile. The temperature range for their growth is 32–37 °C, with an optimum at 35.5 °C. Cells are aerobic and are able to grow on BCYE Cys+, GVPC, and MWY. No growth was observed on non-selective enriched media. The cells’ average size is 0.43 × 1.45 μm. They are positive for oxidase, catalase, and gelatinase, with β-lactamase production. Cells exhibit blue-white autofluorescence under Wood’s lamp (365 nm). The predominant fatty acids are Summed features 3 (C_16:1_ω7c/C_16:1_ω6c), C_16:0_ iso, and C_16:0_, and Q13 is the major ubiquinone. The major lipids are diphosphatidylglycerol (DPG), phosphatidylethanolamine (PE), phosphatidylcholine (PC), and phosphatidylglycerol (PG). Putrescine is the only polyamine detected. The species was isolated from hot water in a healthcare facility water distribution system, located in Bologna, Italy. The type strain 8cVS16^T^ (=DSM 114356^T^ =CCUG 76627^T^) has a G+C content of 38.2 mol%.

## Figures and Tables

**Table 1:** List of main biochemical characteristics of 8cVS16^T^ and 9fVS26 compared with the most related *Legionella* clade and *Lp*1, (+: positive, −: negative).

Species	8cVS16^T^ and 9fVS26	*L. anisa*	*L. bozemanae*	*L. parisiensis*	*L. tucsonensis*	*L. wadsworthii*	*Lp* 1
Accession number	DSM 114356	ATCC 35292	ATCC33217	ATCC 35299	ATCC 40180	ATCC 33877	ATCC 33152
Catalase	+	+	+	+	+	+	+
Urease	−	−	−	−	−	−	−
Hippurate hydrolysis	−	−	−	−	−	−	+
Oxidase	+	+	−	+	−	−	+
β-Lactamase production	+	+	+/−	+	+	+	+
Gelatin liquefaction (gelatinase)	+	+	+	+	+	+	+
Glucose fermentation	−	−	−	−	−	−	−
Autofluorescence	+	+	+	+	+	+	−

**Table 2:** List of main biochemical features of 8cVS16^T^, 9fVS26, *L. anisa* (ATCC 35292^T^), and *Lp*1 (ATCC 33152^T^), (+: positive, −: negative).

Substrates	8cVS16^T^ and 9fVS26	*L. anisa*	*Lp*1	Substrates	8cVS16^T^ and 9fVS26	*L. anisa*	*Lp*1
ARA (arabinose)	−	−	−	GLU (glucose)	−	−	−
MNS (mannose)	−	−	−	PRO (proline-β-naphthylamide)	−	+	−
SUC (sucrose)	−	−	−	PYR (pyrrolidine-b-naphthylamide)	−	−	−
MEL (melibiose)	−	−	−	GGT (g-Glutamyl b-naphthylamide)	−	+	−
RHA (rhamnose)	−	−	−	TRY (tryptophane b-naphthylamide)	−	−	+
SOR (sorbitol)	−	−	−	IND (tryptophane)	−	−	−
MNT (mannitol)	−	−	−	NO_3_ (sodium nitrate)	+	+	+
ADO (adonitol)	−	−	−	GLR (p-nitrophenly β-glucuronide)	−	−	−
ONPG (ρ-Nitrophenyl, b,D-galactoside)	−	−	−	NAG (ρ-Nitrophenyl-N-acetyl-β,D-glucosaminide)	−	−	−
PHO (p-nitrophenyl phosphate)	+	+	+	GGL (γ-L-glutamyl p-nitroanilide)	−	−	−
BGL (p-nitrophenyl α-β-glucoside)	−	−	−	ESC (esculin)	−	−	−
NPG (p-nitrophenyl β-galactoside)	−	−	−	PHE (p-nitro-DL-phenylalanine)	−	−	−
BPH (p-nitrophenyl bis-phosphate)	−	−	−	URE (urea)	−	−	+
BXY (p-nitrophenyl xyloside)	−	−	−	CIT (citrate)	−	−	−
AAR (p-nitrophenyl α-arabinoside)	−	−	−	MLO (malonate)	−	−	−
PHC (p-nitrophenyl phosphorylcholine)	−	−	−	TTC (tetrazolium)	−	−	−
ADH/ARG (arginine)	−	−	−	LYS (lysine)	−	−	−
TRD (aliphatic thiol)	+	+	+	GLY (glycine)	−	−	+
PHS (ρ-Nitrophenyl-phosphoester)	+	+	+	BANA (N-Benzyl-arginine-b-naphthylamide)	−	−	−
αGLU (ρ-Nitrophenyl-α,D-glucoside)	−	−	−	EST (triglyceride)	+	+	+
βGLU (ρ-Nitrophenyl-β,D-glucoside)	−	−	−	INO (inositol)	−	−	−
GAL (galactose)	−	−	−				

+: positive, −: negative.

**Table 3:** Top ten similarity results of genes sequence comparisons among 8cVS16^T^, 9fVS26, and other *Legionella*-type strains officially recognized for 16S rRNA, partial *mip*, and *rpoB* genes. The length of the genes and the accession number are indicated in parentheses.

16S rRNA Gene (1537 bp) (Accession Number)	Identity Percentage	Coverage	*Partial mip* Gene (611 bp) (Accession Number)	Identity Percentage	Coverage	*rpoB* Gene (4107 bp) (Accession Number)	Identity Percentage	Coverage
*L. anisa* DSM 17627^T^ (CP082852.1)	99.29%	100%	*L. anisa* DSM 17627^T^ (CP082852.1)	96.73%	100%	*L. anisa* DSM 17627^T^ (CP082852.1)	95.13%	100%
*L. cherrii* NCTC 11976^T^ (LR134173.1)	98.51%	100%	*L. tucsonensis* ATCC 49180^T^ (U92224.1)	94.95%	100%	*L. cherrii* NCTC 11976^T^ (LR134173.1)	87.60%	100%
*L. sainthelensi* NCTC 11988^T^ (LR134388.1)	98.25%	100%	*L. bozemanae* ATCC 33217^T^ (U91609.1)	94.30%	100%	*L. oakridgensis* NCTC11531^T^ (LR134286.1)	83.94	100%
*L. oakrigensis* NCTC 11531^T^ (LR134286.1)	98.12%	100%	*L. parisiensis* ATCC 35299^T^ (GU083754.1)	93.97%	100%	*L. longbeachae* DSM 10572^T^ (CP082850.1)	83.94%	100%
*L. longbeachae* DSM 10572^T^ (CP082850.1)	98.12%	100%	*L. steigerwaltii* ATCC 35302^T^ (U92223.1)	91.53%	100%	*L. sainthelensi* NCTC11988^T^ (LR134388.1)	83.63%	100%
*L. qingyii* KCTC 15636^T^ (NR_171519.1)	98.80%	97%	*L. cherrii* NCTC 11976^T^ (LR134173.1)	91.35%	99%	*L. lytica* PCM 2298^T^ (CP071527.1)	83.02%	100%
*L. pneumophila* ATCC 33152^T^ (CP040987.1)	97.92%	100%	*L. gormanii* ATCC 33297^T^ (U91638.1)	90.88%	100%	*L. antarctica* NCTC 14581^T^ (AP022839.1)	81.22%	100%
*L. fallonii* ATCC 700992^T^ (LN614827.1)	97.53%	100%	*L. steelei* IMVS 3376^T^ (HQ398203.1)	90.55%	100%	*L. fallonii* DSM 19889^T^ (LN614827.1)	80.91%	100%
*L. parisiensis* JCM 7561^T^ (LC504039.1)	99.05%	95%	*L. wadsworthii* ATCC 33877^T^ (U92225.1)	88.93%	100%	*L. pneumophila subsp. Pascullei* NCTC12273^T^ (LR134380.1)	80.39%	100%
*L. waltersii* NTCT 13017^T^ (LT906442.1)	97.34%	100%	*L. qingyii* KCTC 15636^T^ (MH189580.1)	91.16%	90%	*L. pneumophila* ATCC33152^T^ (CP040987.1)	80.32%	99%

**Table 4:** Genome statistics data from NCBI.

Attribute	Data for Strain	
	8cVS16^T^	9fVS26
No. of raw reads	1,787,078	1,952,986
Avg read length (bp)	256	259
Coverage (×)	115	127
Total Length (bp)	3,906,083	3,906,100
No. of contigs	7	10
GC Content (mol%)	38.2	38.2
N_50_ (bp)	855,940	858,038
No. of coding sequences	3362	3360
No. of rRNAs	6	6
No. of tRNAs	42	42

**Table 5:** Synopsis of the basic genomic data of 8cVS16^T^ and 9fVS26 and the most related *Legionella* species (main clade) and *Lp*1.

Type Strains	Taxon Name	GenBank Accession ID	No. of Contigs	Size (Mbp)	GC (mol%)	No. of CDS	No. of rRNA	No. of tRNA
8cVS16^T^	8cVS16^T^	GCA_021344005.1	7	3.9	38.2	3368	9	42
9fVS26	9fVS26	GCA_021282285.1	10	3.91	38.2	3371	9	42
WA-316-C3	*L. anisa*	GCA_900639785.1	178	4.4	38.17	3.869	3	43
WIGA	*L. bozemanae*	GCA_900640135.1	98	4.13	37.91	3.665	4	43
NCTC11983	*L. parisiensis*	GCA_900461585.1	2	4.2	38.0	3.663	9	44
1087-AZ-H	*L. tusconensis*	GCA_900640035.1	27	3.36	37.41	2.948	3	42
NCTC11532	*L. wadswarthii*	GCA_900452925.1	2	3.6	38.08	3.147	11	43
C9_S	*L. pneumophila*	GCA_001753085.1	3	3.5	38.0	3084	9	43

**Table 6:** MICs of 8cVS16^T^ and 9fVS26 strains and *Lp1* to antimicrobial drugs (gradient MIC method).

Antimicrobial	Concentration Range Tested (mg/L)	MIC for 8cVS16^T^	Interpretation	MIC for 9fVS26	Interpretation	MIC for *Lp*1 ATCC 33152^T^	Interpretation	*Lp* EUCAST Cut-Off (Not Standardized)
Azithromycin	0.016–256	1	R	1	R	0.125	S	0.25
Ciprofloxacin	0.002–32	0.25	S	0.25	S	0.75	S	0.5
Doxicicline	0.016–256	3	S	3	S	N.D.	/	8
Erythromycin	0.016–256	0.5	S	0.5	S	0.125	S	0.5
Levofloxacine	0.002–32	0.125	S	0.125	S	N.D.	/	0.25
Rifampicin	0.002–32	0.064	S	0.064	S	0.023	S	0.032

N.D.: not detected; R: resistant; S: susceptible according to EUCAST guidance document.

**Table 7:** MICs of strain 8cVS16^T^, 9fVS26, and *Lp*1 to antimicrobial drugs (microbroth dilution method).

Antimicrobial	Concentration Range Tested (mg/L)	MIC for 8cVS16^T^	Interpretation	MIC for 9fVS26	Interpretation	MIC for *Lp1* ATCC 33152^T^	Interpretation	*Lp* EUCAST Cut-Off (Not Standardized)
Azithromycin	0.0075–16	1	R	1	R	0.5	R	0.125
Erythromycin	0.0075–16	0.5	S	0.5	S	1	S	1
Ciprofloxacin	0.00025–0.512	0.016	S	0.016	S	0.032	S	0.032

R: resistant; S: susceptible according to EUCAST guidance document.

## Data Availability

The GenBank accession numbers for the strains 8cVS16^T^ and 9fVS26 are as follows:16S rRNAs: OL804581.1 and OL889882.1; *mip*: MW052957.1 and MW052913.1; *rpoB*: MZ367138 and MZ367095.The GenBank accession numbers for the whole genome sequencing of strains 8cVS16^T^ and 9fVS26 are JAJTND000000000 and JAJSPM000000000, respectively.

## Supplementary Materials

The following supporting information can be downloaded at: https://www.mdpi.com/article/10.3390/pathogens13030250/s1, Figure S1: The 8cVS16^T^ and 9fVS26 strains’ growth evaluation on different media: (1) GVPC, (2) MWY, (3) BCYE Cys+, and (4) Cys−, (5) TSA Blood Agar, and (6) Chocolate Enriched Agar; Figure S2: Dendrogram based on whole-cell MALDI-TOF mass spectra for the strains 8cVS16^T^ and 9fVS26 and *Legionella* reference strains present in the instrument database; Figure S3: Lipid contents of 8cVS16^T^ and 9fVS26 (A), and *L. anisa* (DSM 17627^T^) (B); Figure S4: Phylogenetic tree based on *mip* gene of the two strains (8cVS16^T^ and 9fVS26) and closely related species of the genus *Legionella*. Branch labels display the substitutions per site. The bootstrap values are 100, using the “Rapid” bootstrapping option of RaxML. Bar 0.007 substitution per nucleotide position. The strains 8cVS16^T^ and 9fVS26 are highlighted in red; Figure S5: Phylogenetic tree based on *rpoB* gene of the two strains (8cVS16^T^ and 9fVS26) and closely related species of the genus *Legionella*. Branch labels display the substitutions per site. The bootstrap values are 100, using “Rapid” bootstrapping option of RaxML. Bar 0.02 substitution per nucleotide position. The strains 8cVS16^T^ and 9fVS26 are highlighted in red; Figure S6: Phylogenetic tree based on the 16S rRNA gene of the two strains (8cVS16^T^ and 9fVS26) and closely related species of the genus *Legionella*. Branch labels display the substitutions per site. The bootstrap values are 100, using the “Rapid” bootstrapping option of RaxML. Bar 0.002 substitution per nucleotide position. The strains 8cVS16^T^ and 9fVS26 are highlighted in red; Figure S7: REP-PCR DNA and BOX PCR fingerprinting products for the strains 8cVS16^T^ (1) and 9fVS26 (2) visualized by electrophoresis gel 2% *w*/*v* to assess the isolates’ clonality. The gel represents the following: section A, the gene products of both strains amplified by REP primers: lane 1 for 8cVS16^T^ and 2 for 9fVS26 at a DNA concentration of 70 ng, and lane 3 for 8cVS16^T^ and 4 for 9fVS26 as DNA “undiluted”; section B, the gene product obtained by BOX primer amplification: lines 5 and 6 for 8cVS16^T^ and 9fVS26, respectively, at a DNA concentration of 70 ng, and lines 7 and 8 for 8cVS16^T^ and 9fVS26 as DNA “undiluted”; section C, lane 9 positive (C+) control for REP-PCR, lane 10 positive (C+) control for BOX-PCR, lane 11 negative (C−) control for REP-PCR, and lane 12 negative (C−) control for BOX-PCR. Lane “L”: reference marker sizes in base pairs (1kb); Figure S8: Genome alignment of *L. anisa* (ATCC 35292^T^), 8cVS16^T^, 9fVS26, and *Lp*1 (ATCC 33152^T^), provided by BLAST Ring Image Generator (v. 0.95) software; Table S1: List of 63 *Legionella* species, plus 8cVS16^T^ and 9fVS26, utilized for the genome data comparison (continued); Table S2: Cellular fatty acid (CFA) composition of 8cVS16^T^, 9fVS26, and most-related *Legionella* species: *L. anisa* (DSM 17627^T^), *L. bozemanae* (ATCC 33217^T^), *L. parisiensis* ATCC 35299^T^), *L. tucsonensis* (ATCC 40180^T^), *L. wadsworthii* (ATCC 33877^T^), and *L. pneumophila* subsp. *pneumophila* Philadelphia 1 (ATCC 33152^T^). (-), Not detected; NA, not available; Tr, traces. The values indicate the percentages of total fatty acids found; Table S3: Ubiquinones contents of 8cVS16^T^, 9fVS26, most-related *Legionella* species, and *Lp*1^a^; Table S4: Comparison among 8cVS16^T^ and 9fVS26 strains, *L. anisa* (ATCC 35292^T^), and *L. pneumophila philadelphia* Serogroup 1 (ATCC 33152^T^); list of missing genes. Table S5. List of virulence genes shared among the Legionella species belonging to the same clade: 8cVS16T, 9fVS26, *Legionella anisa* WA-316-C3 ATCC35292T, *Legionella bozemanae* WIGA ATCC 33217T, *Legionella parisiensis* PF-209C-C2 ATCC35299T, *Legionella tusconensis* 1087AZH ATCC49180T, *Legionella wadsworthii* 81-716A ATCC 33877 T. [26,27,28,39,40].
