# Supplementary material for: Characterization of a Novel Species of Legionella Isolated from a Healthcare Facility: Legionella resiliens sp. nov
Source: Pathogens. 2024 Mar 14;13(3):250. doi: 10.3390/pathogens13030250 (PMC10975825; doi:10.3390/pathogens13030250)
Supplement: Supplementary file 1 [file pathogens-13-00250-s001.zip › pathogens-2865926-supplementary.pdf]

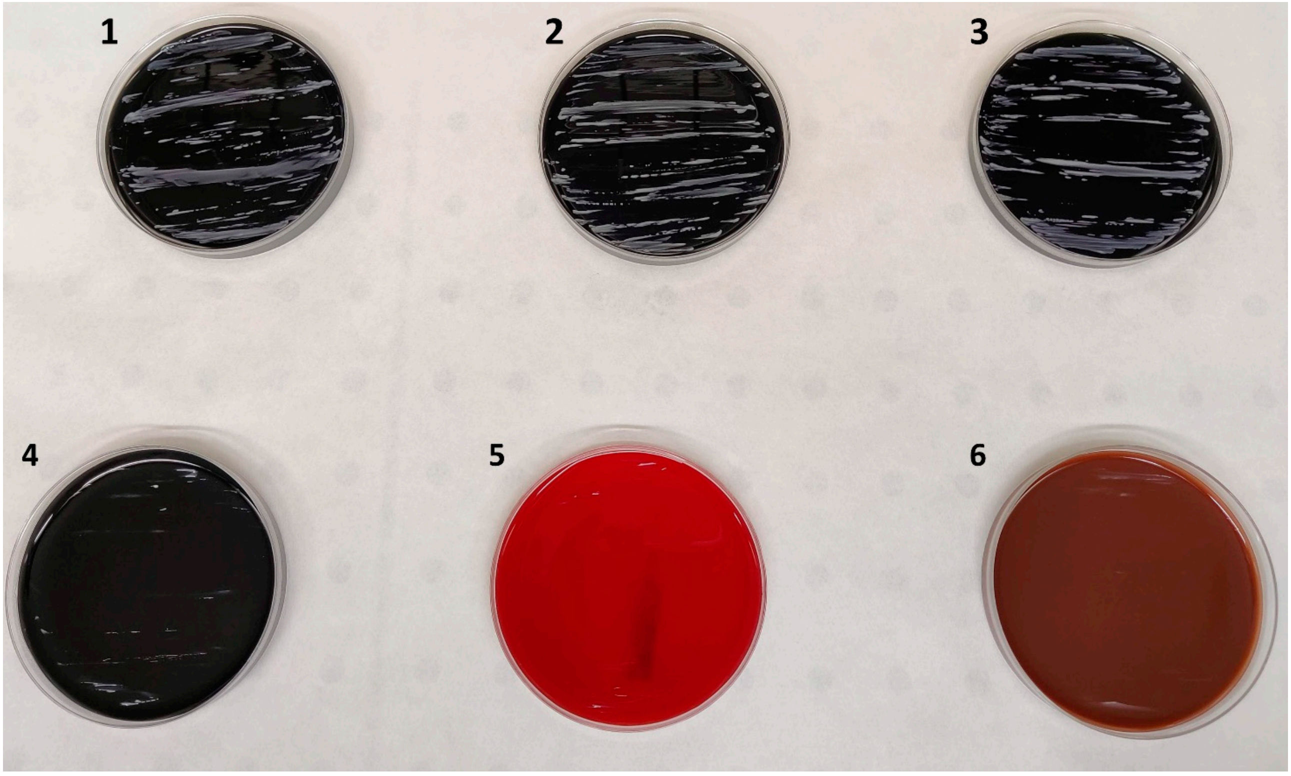

Figure S1. The strain 8cVS16^{T} and 9fVS26 growth evaluation on different media: (**1**) GVPC, (**2**) MWY, (**3**) BCYE Cys+ and (**4**) Cys-, (**5**) TSA Blood Agar and (**6**) Chocolate Enriched Agar.

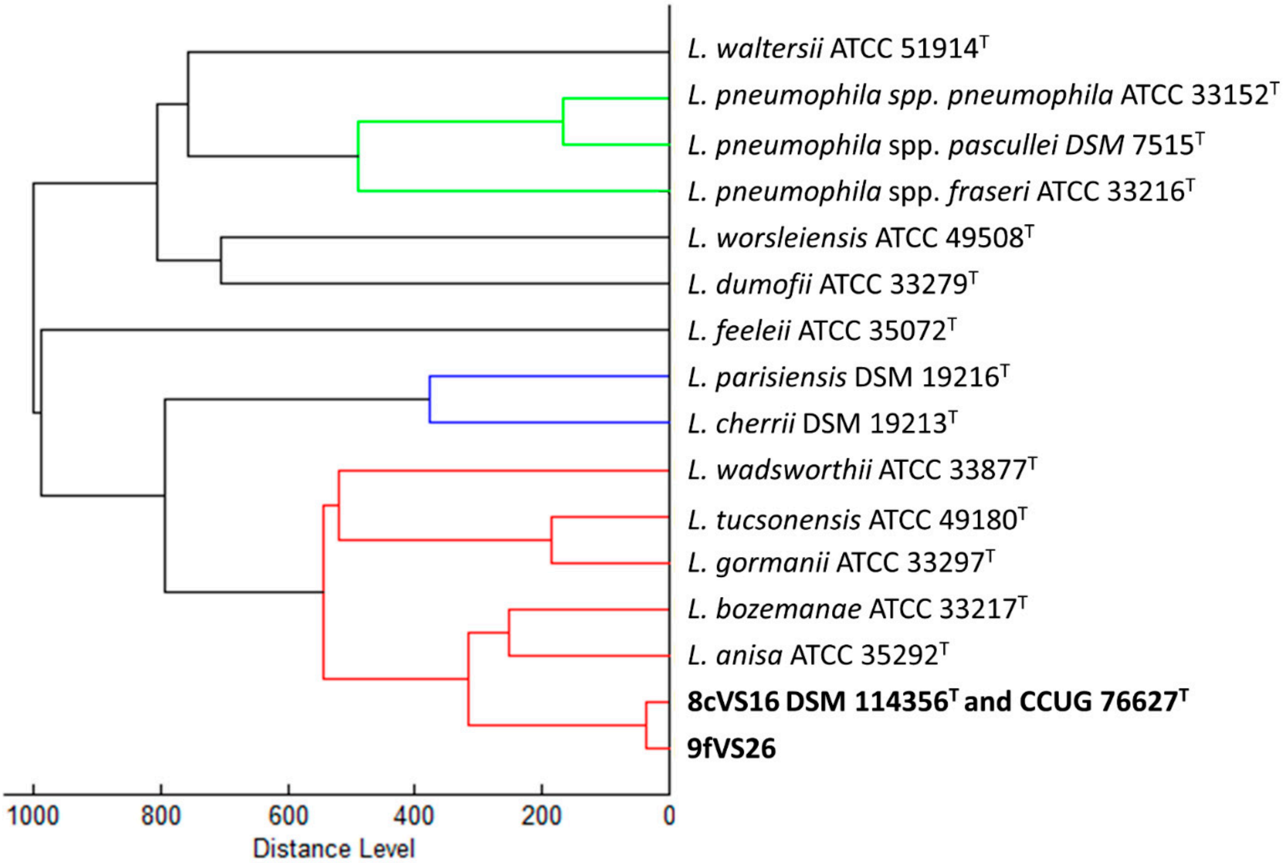

Figure S2. Dendrogram based on whole-cell MALDI-TOF mass spectra for the strains 8cVS16^{T} and 9fVS26 and *Legionella* reference strains present in instrument database.

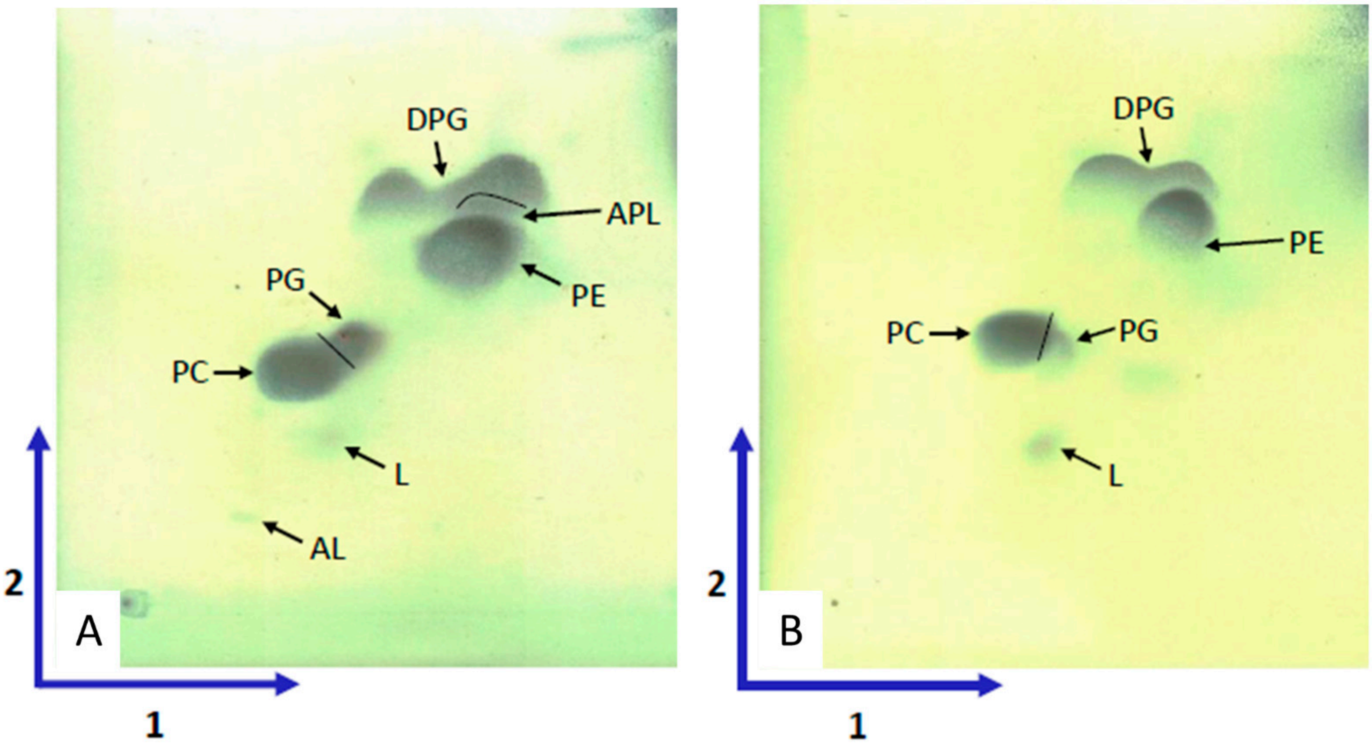

Figure S3. Lipids contents of 8cVS16^T and 9fVS26 (**A**), and *L. anisa* (DSM 17627^T) (**B**).

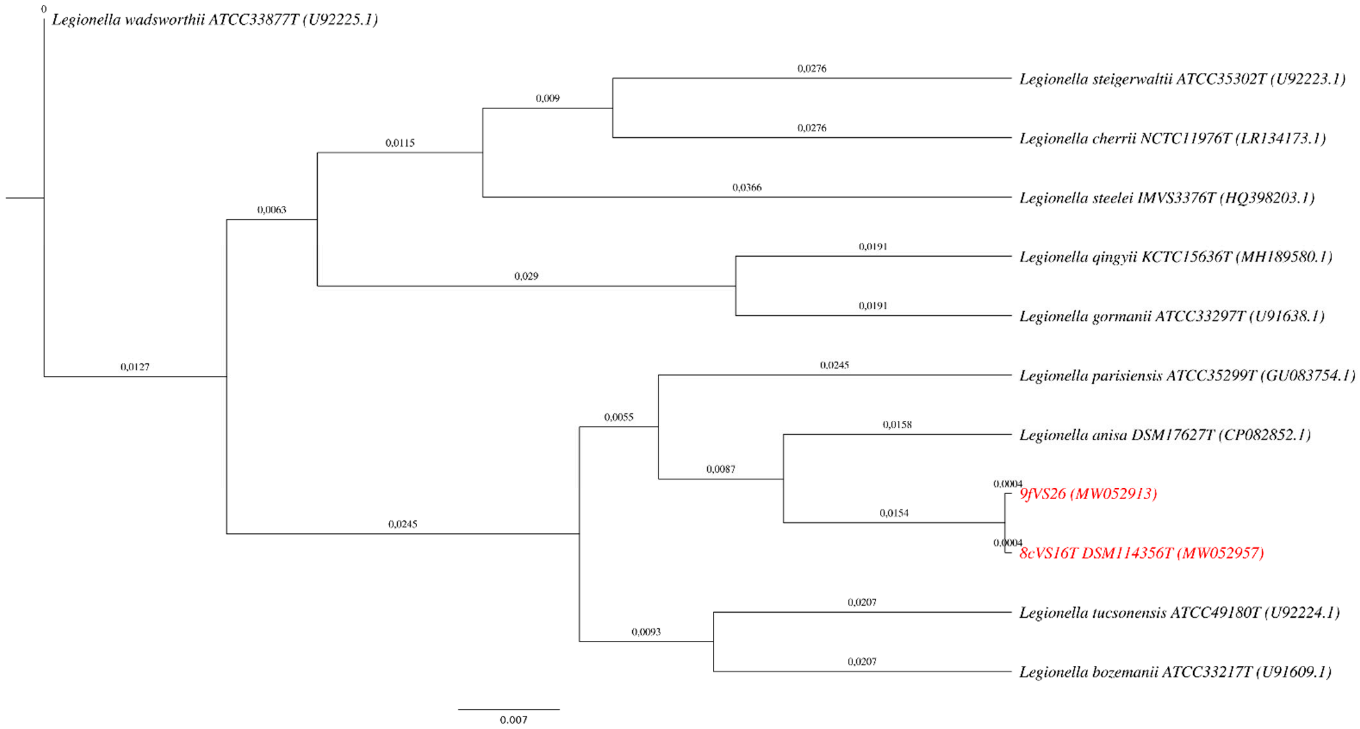

Figure S4. Phylogenetic tree based on *mip* gene of the two strains (8cVS16^T and 9fVS26) and closely related species of the genus *Legionella*. Branch labels show substitutions per site calculated by Bayesian inference using the Markov Chain Monte Carlo (MCMC) method [39,40]. Bar 0.007 substitution per nucleotide position. The strains 8cVS16^T and 9fVS26 are highlighted in red.

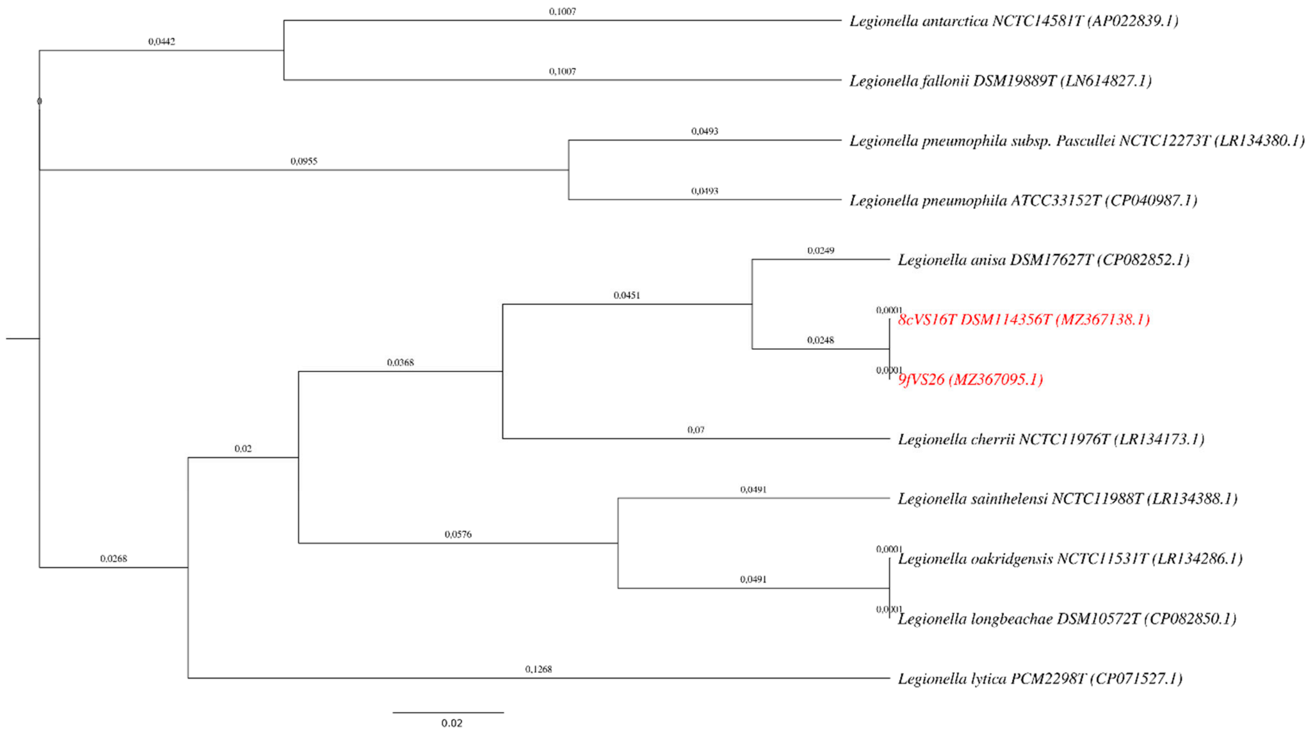

Figure S5. Phylogenetic tree based on *rpoB* gene of the two strains (8cVS16^T and 9fVS26) and closely related species of the genus *Legionella*. Branch labels show substitutions per site calculated by Bayesian inference using the Markov Chain Monte Carlo (MCMC) method [39,40]. Bar 0.02 substitution per nucleotide position. The strain 8cVS16^T and 9fVS26 are highlighted in red.

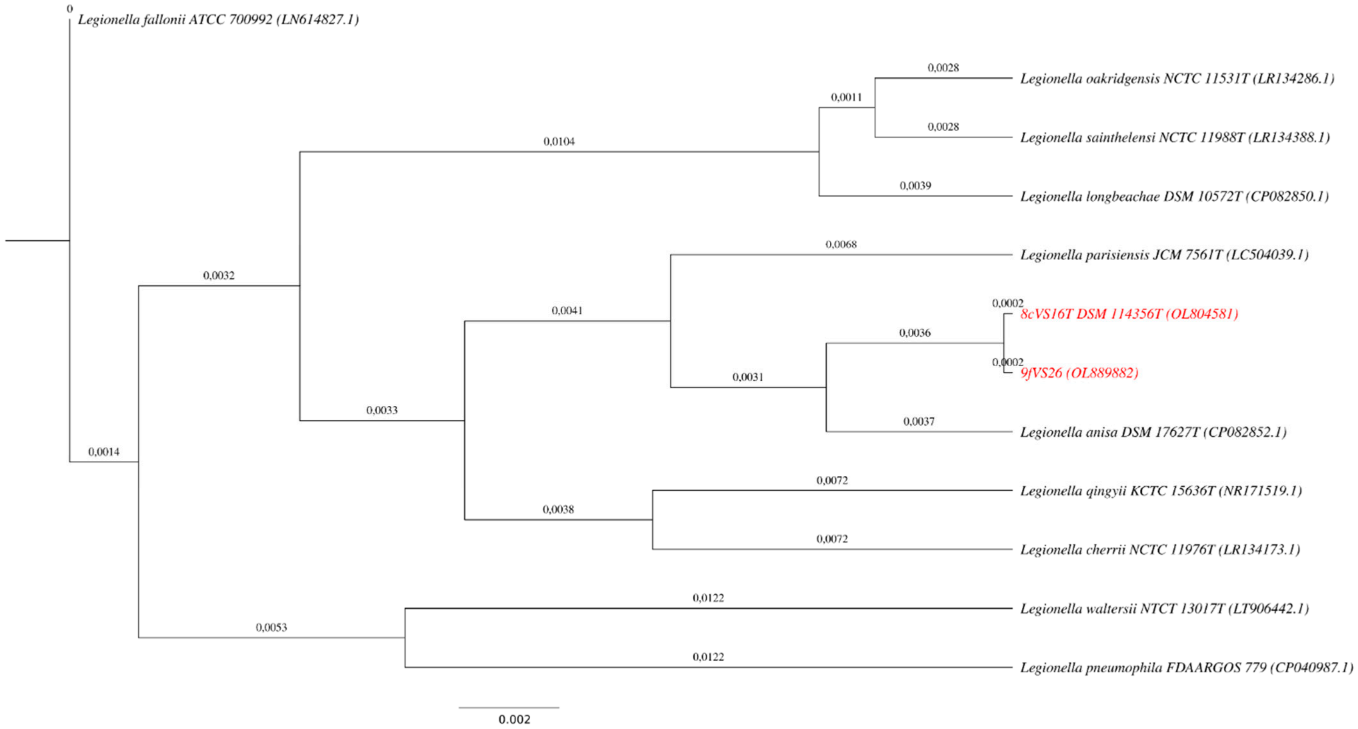

.

Figure S6. Phylogenetic tree based on 16S rRNA gene of the two strains (8cVS16^T and 9fVS26) and closely related species of the genus *Legionella*. Branch labels show substitutions per site calculated by Bayesian inference using the Markov Chain Monte Carlo (MCMC) method [39,40]. Bar 0.002 substitution per nucleotide position. The strain 8cVS16^T and 9fVS26 are highlighted in red.

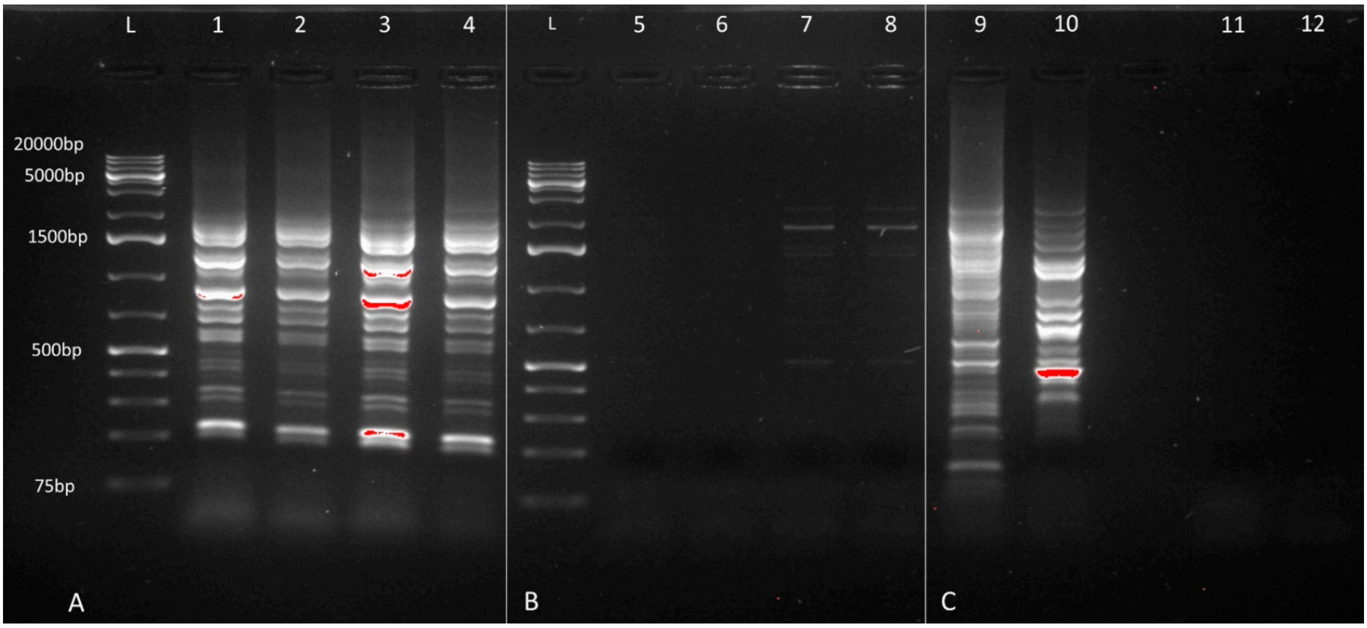

Figure S7. REP-PCR DNA and BOX PCR fingerprinting products for the strains 8cVS16^T (1) and 9fVS26 (2) visualized by electrophoresis gel 2% w/v, to assess the isolates clonality. The gel represents: section **A**, the gene products of both strain amplified by REP primers: lane 1 for 8cVS16^T and 2 for 9fVS26 at DNA concentration of 70 ng; lane 3 for 8cVS16^T and and 4 for 9fVS26 as DNA "undiluted"; section **B**, the gene product obtained by BOX primer amplification: lines 5 and 6 for 8cVS16^T and 9fVS26, respectively at a DNA concentration of 70 ng; lines 7 and 8 for 8cVS16^T and 9fVS26 as DNA "undiluted"; section **C,** lane 9 positive (C+) control for REP-PCR, lane 10 positive (C+) control for BOX-PCR, lane 11 negative (C-) control for REP-PCR and lane 12 negative (C-) control for BOX-PCR. Lane "L": reference marker sizes in base pairs (1kb).

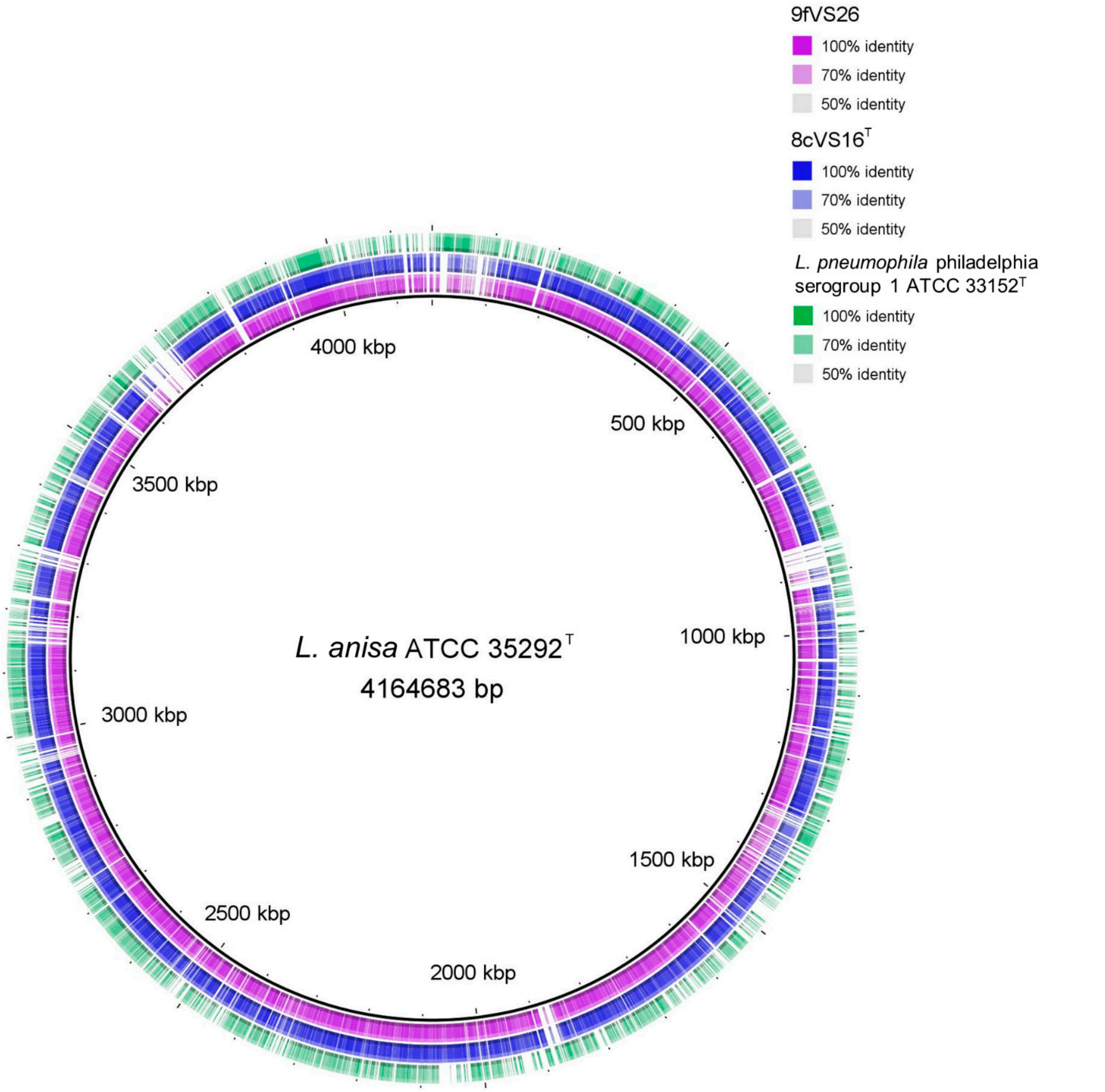

Figure S8. Genome's alignment of *L. anisa* (ATCC 35292^T), 8cVS16^T, 9fVS26 and *Lp*1 (ATCC 33152^T), provided by BLAST Ring Image Generator (v. 0.95) software.

Table S1. List of 63 *Legionella* species, plus 8cVS16^T and 9fVS26, utilized for the genome data comparison.

Genome Name	Strain	Culture Collection	Assembly Accession	GenBank Accessions
**8cVS16**	8cVS16	DSM:114356	GCA_021344005.1	JAJTND000000000.1
**9fVS26**	9fVS26		GCA_022182285.1	JAJSPM000000000.1
Fluoribacter dumoffii	NY 23	ATCC:33279	GCA_900450695.1	UGGT00000000.1
Fluoribacter gormanii	LS-13	ATCC:33297	GCA_001467685.1	LNYD00000000.1
Legionella adelaidensis	1762-AUS-E	ATCC:49625	GCA_001467055.1	LNKA00000000.1
Legionella anisa	WA-316-C3	ATCC:35292	GCA_900639785.1	CAAAHR000000000.1
Legionella antarctica	TUM19329	NCTC:14581	GCA_011764505.1	AP022839.1
Legionella beliardensis	Montbéliard A1	ATCC:700512	GCA_900452395.1	UGNV00000000.1
Legionella birminghamensis	1407-AL-H	ATCC:43702	GCA_900452515.1	UGNW00000000.1
Legionella bononiensis	30cS62	ATCC:TSD-262	GCA_016786415.1	JADWVN000000000.1

Legionella bozemanae	WIGA	ATCC:33217	GCA_900640135.1	CAAAIY000000000.1
Legionella brunensis	441-1	ATCC:43878	GCA_001467025.1	LNXV00000000.1
Legionella busanensis	K9951	ATCC:BAA-518	GCA_900461525.1	UGOD00000000.1
Legionella cardiaca	H63	ATCC:BAA-2315	GCA_029026145.1	CP119078.1
Legionella cherrii	ORW	ATCC:35252	GCA_000621385.1	JHYM00000000.1
Legionella cincinnatiensis	72-OH-0	ATCC:43753	GCA_001467545.1	LNXX00000000.1
Legionella drancourtii	LLAP12	ATCC:50991	GCA_000162755.2	ACUL00000000.2
Legionella drozanskii	LLAP-1	ATCC:700990	GCA_900640075.1	CAAAIU000000000.1
Legionella erythra	SE-32A-C8	ATCC:35303	GCA_001467615.1	LNYA00000000.1
Legionella fairfieldensis	1725-AUS-E	ATCC:49588	GCA_900640125.1	CAAAIZ000000000.1
Legionella fallonii	LLAP-10	ATCC:700992	GCA_000953135.1	LN614827.1
Legionella feeleii	WO-44C	ATCC:35072	GCA_001467625.1	LNYB00000000.1
Legionella geestiana	1308	ATCC:49504	GCA_001467645.1	LNYC00000000.1
Legionella gratiana	Lyon 8420412	ATCC:49413	GCA_001467695.1	LNYE00000000.1
Legionella gresilensis	Gréoux 11 D13	ATCC:700509	GCA_900639865.1	CAAAHX000000000.1
Legionella hackeliae	Lansing 2	ATCC:35250	GCA_000953655.1	LN681225.1
Legionella impletisoli	OA1-1	DSM:18493	GCA_900639875.1	CAAAIA000000000.1
Legionella israelensis	Bercovier 4	ATCC:43119	GCA_004571175.1	CP038273.1
Legionella jamestowniensis	JA-26-G1-E2	DSM:19215	GCA_900114725.1	FOTZ00000000.1
Legionella jordanis	BL-540	NCTC:11533	GCA_900637635.1	LR134383.1
Legionella lansingensis	1677-MI-H	NCTC:12830	GCA_900187355.1	LT906451.1
Legionella londiniensis	1477	ATCC:49505	GCA_900452755.1	UGON00000000.1
Legionella longbeachae	Long Beach 4	ATCC:33462	GCA_004283175.1	RXNW00000000.1
Legionella lytica	L2	PMC:2298	GCA_023921225.1	CP071527.1
Legionella maceachernii	PX-1-G2-E2	ATCC:35300	GCA_900460175.1	UHIB00000000.1
Legionella maioricensis	HCPI-6	CCUG:75071	GCA_023618015.1	JAJKBJ000000000.1
Legionella massiliensis	LegA	DSM:24804	GCA_000756815.1	CCVW00000000.1
Legionella moravica	316-86	ATCC:43877	GCA_900452715.1	UGOG00000000.1
Legionella nagasakiensis	CDC-1796-JAP-E	ATCC:BAA-1557	GCA_900639915.1	CAAAID000000000.1
Legionella nautarum	1224	ATCC:49506	GCA_001467895.1	LNYO00000000.1
Legionella norrlandica	LEGN	ATCC:BAA-2678	GCA_000770585.1	JNCF00000000.1
Legionella oakridgensis	Oak Ridge 10	ATCC:33761	GCA_001648605.1	LCUA00000000.1
Legionella parisiensis	PF-209C-C2	ATCC:35299	GCA_900461585.1	UGOH00000000.1
Legionella pneumophila	Philadelphia-1	ATCC:33152	GCA_001941585.1	CP013742.1
Legionella qingyii	km488	KCTC:15636	GCA_003184185.1	QHJG01000001.1
Legionella quateirensis	1335	ATCC:49507	GCA_900452695.1	UGOW00000000.1
Legionella quinlivanii	1442-AUS-E	ATCC:43830	GCA_001467975.1	LNYS00000000.1
Legionella rowbothamii	LLAP6	ATCC:700991	GCA_900639985.1	CAAAIM000000000.1
Legionella rubrilucens	WA-270A-C2	ATCC:35304	GCA_001468125.1	LNYT00000000.1
Legionella sainthelensi	MSH-4	ATCC:35248	GCA_001468105.1	LNYV00000000.1
Legionella santicrucis	SC-63-C7	ATCC:35301	GCA_001468135.1	LNYU00000000.1
Legionella saoudiensis	LS-1	DSM:101682	GCA_001465875.1	CZVG00000000.1
Legionella septentrionalis	km711	KCTC:15655	GCA_003989745.1	RZGS00000000.1
Legionella shakespearei	214	ATCC:49655	GCA_001468025.1	LNYW00000000.1
Legionella spiritensis	ML76	NCTC:12082	GCA_900637495.1	LR134374.1
Legionella steelei	IMVS-3376	ATCC:BAA-2169	GCA_001468005.1	LNYY00000000.1
Legionella steigerwaltii	SC-18-C9	ATCC:35302	GCA_900452835.1	UGOY00000000.1
Legionella taurinensis	Turin I no 1	ATCC:700508	GCA_900452865.1	UGOZ00000000.1
Legionella tucsonensis	1087-AZ-H	ATCC:49180	GCA_900640035.1	CAAAIP000000000.1
Legionella tunisiensis	LegM	DSM:24805	GCA_000308315.1	CALJ00000000.1
Legionella wadsworthii	Wadsworth 81-716A	ATCC:33877	GCA_900452925.1	UGPB00000000.1
Legionella waltersii	2074-AUS-E	ATCC:51914	GCA_900187095.1	LT906442.1
Legionella worsleiensis	1347	ATCC:49508	GCA_900453045.1	UGPA00000000.1
Legionella yabuuchiae	OA1-2	DSM:18492	GCA_900640115.1	CAAAIW000000000.1
Tatlockia micdadei	TATLOCK	ATCC:33218	GCA_000953635.1	LN614830.1

Table S2. Cellular fatty acid (CFA) composition of 8cVS16^T, 9fVS26 and *Legionella* most related species: *L. anisa* (DSM 17627^T), *L. bozemanae* (ATCC 33217^T), *L. parisiensis* ATCC 35299^T), *L. tucsonensis* (ATCC 40180^T), *L. wadsworthii* (ATCC 33877^T) and *L. pneumophila subsp. pneumophila* Philadelphia 1 (ATCC 33152^T). (-), not detected; NA, not available; Tr, traces. The values indicate the percentages of total fatty acids found.

Fatty acid	8cVS16^T and 9fVS26	*L. anisa*	*L. bozemanae*[a]	*L. parisiensis*[a]	*L. tucsonensis*[b]	*L. wadsworthii*[a]	*L. pneumophila*[c]
**Saturated**							
$C_{14:0}$	1.5	0.4					-
$C_{15:0}$	3.1	1.8					Tr
$C_{16:0}$	16.3	11.5	11.0	9.0	10.0	6.0	12.0
$C_{16:1}$					20.0		
$C_{17:0}$	1.1	2.0			1.0		1.0
$C_{18:0}$	0.8	1.5	2.0	1.0	1.0	1.0	3.0
$C_{19:0}$	0.3	0.3	1.0				
$C_{20:0}$	2.1	0.5	1.0				2.0
**Unsatured**							
$C_{14:1}$ ω5*c*	1.0	0.3					Tr
$C_{15:1}$ ω6*c*	3.4	2.1					1.5
$C_{16:1}$ ω7*c*	0.5		9.0			8.0	29.6
$C_{16:1}$ ω11*c*	1.0			7.0			
**Cyclopropane**							
$C_{16:0}$ cyclo					1.0		
$C_{17:0}$ cyclo	0.4	0.8	9.0	16.0	7.0	6.0	-
**Methyl branched (iso, anteiso)**							
$C_{15:0}$ anteiso	14.2	29.2	29.0	23.0	25.0	37.0	9.4
$C_{15:1}$ anteiso A		0.8					
$C_{17:0}$ anteiso	4.4	9.7	11.0	9.0	12.0	19.0	7.4
$C_{19:0}$ anteiso						2.0	
$C_{14:0}$ iso	2.8	2.7	3.0	3.0	2.0	1.0	2.4
$C_{15:0}$ iso	0.5	0.3	tr			Tr	
$C_{16:0}$ iso	17.5	14.7	14.0	17.0	20.0	10.0	23.4
$C_{17:0}$ iso	0.5	0.6	1.0	1.0		1.0	
$C_{18:0}$ iso	0.2	0.2					
**Branched-chain hydroxy**							
$C_{14:0}$ iso 3OH	0.5				2.0		Tr
$C_{15:0}$ iso 2OH	-				1.0		-
$C_{16:0}$ iso 3OH		0.1			13.0		
**Summed features**							
2 ($C_{16:1}$ iso I/ $C_{14:0}$ 3OH)	0.2	0.1					
3 ($C_{16:1}$ω7c/$C_{16:1}$ω6 c)	27.7	20.5					

Except for 8cVS16^T, 9fVS26 and *L. anisa* (DSM17627^T), the strains information was obtained from literature: [a]Lambert and Moss, 1988 [27] [b]Thacker et al., 1989 [28]; [c]Crespi et al., 2023 [26].

Table S3. Ubiquinones contents of 8cVS16^T, 9fVS26, *Legionella* most related species and *Lp*1[a].

Species[b]	Accession number	Q-9	Q-10	Q-11	Q-12	Q-13	Q-14	Q-15
8cVS16^T and 9fVS26	DSM 114356 ^{T}			0.70%		50.20%	39.60%	1.20%
L. anisa	DSM 17627 ^{T}	12.60%	33.00%	24.20%	26.90%	2.90%	0.40%	
L. bozemanae	ATCC 33217 ^{T}	2-3	4	3-4	4	tr-1	-	
L. parisiensis	ATCC 35299 ^{T}	1-2	3-4	3	4	1	-	
L. tucsonensis	ATCC 40180 ^{T}				NA			
L. wadsworthii	ATCC 33877 ^{T}	2	4	2-3	tr-1	-	-	
L. pneumophila	ATCC 33152 ^{T}	-	-	1-2	4	1-2	-	

[a] The numbers indicate the visual estimates of the relative amounts of ubiquinone with the major component labeled as 4; 2, half the amount of 4; 1, half that of 2; -, not detected; NA, not available; tr, traces. Differences between strains within a species are indicated by ranges. [b] Except 8cVS16^T, 9fVS26 and *L. anisa*, the strains information was obtained from Lambert et al. 1988 [27].

Table S4. Comparison among 8cVS16^T and 9fVS26 strains, *L. anisa* (ATCC 35292^T) and *L. pneumophila philadelphia* Serogroup 1 (ATCC 33152^T): list of missing genes.

Genome comparison	Total number of annotated genes	List of missing genes in shorter genome
9fVS26<br>*vs*<br>8cVS16^T	1596<br>*vs*<br>1595	**9fVS26 genes not found in 8cVS16:** *esiB_1, esiB_2*
		**8cVS16 genes not found in 9fVS26:** *esiB*
8cVS16^T<br>*vs*<br>*L. anisa*	1595<br>*vs*<br>1677	**8cVS16 genes not found in *L. anisa*:** *murU_1, murU_2, sdh, psuK, mdtK, pepA, alx_1, glaR, potA, potB, potD, nat, glsA1, ackA, ytcD, ycdF, tolB_1, cbpD, rssB_1, ypeA, aacA4_1, rssB_2, yhcG, hin, kmo_1, aatB_1, purE, cheW, cheR1, cyaB_1, nprV, rcsB, cph1, dauR, atpC, atpD, atpG, atpA, atpF, atpB, recF, cbs, panD, guaC, hldD, cspC, rocG_1, ybaB_1, mhqR, petC_1, petC_2, sspA, ydgA, hrp1_1, ligd, sufS, algC, doeB, rhtC_1, kmo_2, yhaJ, aspC, adhT, cadI_1, nhaP, asnB, treA, gloB_1, pafA_1, rcsA, ctpF, uspE_1, nhaA, acr3, arsC, cadI_2, nfdA, ycaC_1, dnaG_1, bioC, phrA, lolE, ldh, pagN, dps2_1, motA, adc_1, aacA4_2, tdcD, rocG_2, yhjQ, ftsP_1, yhhT, aatB_2, cysC, pcp, panE, hspA, alkA, mtnX, gshA, ykfA_3, dhaA_1, argP, hutG, lcfB_1, pdaC, ycaC_2, ftsI, gtaB, yhjR, bcsQ, yabJ_1, ybaB_2, tadA_1, gltP_1, lexA, ligD, ku_1, aacA4_3, doxA, yrbG, hoxH, asrB, ndhI, hypE, hypD, hypC, hypF, hypB, nixA, uspE_2, pacL, auaG, nqrF, cyaB_2, bepA, fabI_4, pafA_2, macA, fcl, pds, arnC_3, rfbC, garP, recB, dprE1, udk_3, qseC, gloB_2, emrA_1, yyaP_1, clcA_3, lacG, guaB, tadA_2, mscS, yyaP_2, ybaB_3, htrA, hpf_1, ydaD, tolB_2, derI, yveA, ibpA, glpE, ftsP_2, pah, hrp1_2, sthA, ynjF, yabJ_2, dadA, bioD1, bioH_1, cmk, thiK, fabH, esiB, dnaG_2, intA, dhaA_2, golD, bioH_2, pksC, xerC, gstB, gbpA, atm1, aaeB, korB, atoC_3, dauA, ampG_1, tam_3, gdhA, alx_2, rlpA_3, malT, adc_2, ankX, arcB_3, lcfB_2, todT_3, phaB_5, ampG_2, spxA, gltP_2, kdpB_4, sasA_10, ku_2, rcsC_6, mnaT, csdA, vldW, carA_5, arnC_4, yceF, dps2_2, lcfB_3, rhtC_2, hpf_2, ahpD, sasA_11, hemH, csbC, edd, pgl, gadC_4, ybhR_1, btuD_3, ybhR_2, emrA_2, sadH, lemA, oprM_5, oprM_6, murU_3*
		***L. anisa* genes not found in 8cVS16:** *catD_1, malT_1, ackA_1, xpkA_1, atpD_1, atpC_1, atpB_1, atpF_1, atpA_1, atpG_1, ftsP, esiB_1, cusA, cusB, lexA_1, cpbD_1, xerC_1, xerC_2, xerC_3, xerC_4, yhaJ_1, kmo, ygiD, algC_1, ldh_1, sufS_1, sspB, sspA_1, petC, petA, hemX, ybaB, rocG, fcl_1, guaB_1, yhaJ_2, panD_1, cysK, recF_1, osmY, atpB_2, atpF_2, atpH_3, atpA_2, atpG_2, atpD_2, atpC_2, glpQ, catD_2, cmpR, tagH, epsL, epsM, per, mhpC, mta, cyaB, traM_1, traL_1, traK_1, traJ_1, traI_1, traG_1, virB4_1, lexA_2, egtB_1, egtB_2, gshA_1, hemH_1, purK_1, purE_1, ndhB_1, gltR, apxIB_1, edd_1, glsA1_1, bioD1_1, phrA_1, crtK-2, phrB, ufaA1, purK_2, purE_2, ndhB_2, apxIB_2, panD_2, ddl, ygeA, mdrP, rizA, xpkA_2, aacA4, lon2, ephD, bcr, rimL, paaK, sufS_2, glaH, gshA_2, livK, rssB, csbC_1, kdnA, neoG, csbC_2, yveA_1, ohrA, adh, cda, cspV, dnaG, lolE_1, ldh_2, dps2, motA_1, recF_2, xerC_5, xerD_1, xerD_2, wecD_1, csrA_3, thiK_1, desA, baeB, baeC_1, novP, lolE_2, hin_1, lexA_3, aatB, cmk_1, yofA, glaR_1, fixL, cheB_3, ibpA_1, serA, xerD_3, xerD_4, xerC_6, gshA_3, rimJ, dhaA, srkA_3, ttuB, hutG_1, lcfB, htrA_1, ycaC, ftsI_1, gtaB_1, yabJ, mmcO, motA_2, xerC_7, xerD_5, xerD_6, xerC_8, xerD_7, xerD_8, ahpD_1, argT, bepA_1, pafA, pgl_1, spsI, fcl_2, dadA_1, mshA_3, rmlC, gudP, addA, dprE1_1, motB_3, tylM1, mltD, gloB, emrA, yyaP, fadA_3, add, araQ, guaB_2, tadA, gltC, hchA, cvfB, cspA_3, tolB, dltA_3, rpiB, yveA_2, ibpA_2, copB, copA_3, hutG_2, hrp1, thi4, slrP, dadA_2, yajO, bioD1_2, bioH, cmk_2, thiK_2, oprB, fabH_1, esiB_2, baeC_2, xerC_9, dgt, gstA, bioD1_3, glsA1_2, edd_2, besD, ccmH, dauA_1, ampG, sfp, csrA_4, dtpA_4, pal_3, dauA_2, phrA_2, ibpB, htrA_2, spkD, dprE1_2, malT_2, prs_3, cadA, adc, sspA_2, intS_3, csrA_5, celA, gtaB_2, algC_2, wbiB, bepA_2, galE1, ywqF, yfcJ, rayT, yfgD, gltP, ligD_1, ligD_2, ku, mshD_3, tycC, maf, lexA_4, mrgA, hin_2, dltA_4, fabG_6, mepM, spmB, spmA, pepA_1, holC, pepA_2, murJ, rpsT, uvrC, gacA, phhA, cinA, obg, rpmA, rplU, rplY, pth, ychF, ispB, cpbD_2, proP_6, esiB_3, traM_2, traL_2, traK_2, traJ_2, traI_2, traD, traG_2, virB4_2, csrA_6, xerC_10, ftsI_2, mecI, thrB, rhtC, hpf, smc_6, secA_3, ahpD_2, wecD_2, hemH_2, ppaX, csbC_3, edd_3, pgl_2, fabH_2, oleD, ahlD, ttgC, acpP_5, murU, ydfG, pepA_3, alx, frsA, glaR_2, tmoT, fosB, glsA, ackA_2, srfAB*
9fVS26<br>*vs*<br>*L. anisa*	1596<br>*vs*<br>1677	**9fVS26 genes not found in *L. anisa*:** *murU_1, murU_2, sdh, psuK, mdtK, pepA, alx_1, glaR, potA, potB, potD, nat, glsA1, ackA, ytcD, ycdF, tolB_1, cbpD, rssB_1, ypeA, aacA4_1, rssB_2, yhcG, hin, kmo_1, aatB_1, purE, cheW, cheR1, cyaB_1, nprV, rcsB, cph1, dauR, atpC, atpD, atpG, atpA, atpF, atpB, recF, cbs, panD, guaC, hldD, cspC, rocG_1, ybaB_1, rhtC_1, hpf_1, ahpD, hemH, csbC, edd, pgl, ybhR_1, ybhR_2, emrA_1, sadH, lemA, murU_3, mhqR, petC_1, petC_2, sspA, ydgA, hrp1_1, ligd, sufS, algC, doeB, rhtC_2, kmo_2, yhaJ, aspC, adhT, cadI_1, nhaP, asnB, treA, gloB_1, pafA_1, rcsA, ctpF, uspE_1, nhaA, acr3, arsC, cadI_2, nfdA, ycaC_1, dnaG_1, bioC, phrA, lolE, ldh, pagN, dps2_1, motA, adc_1, aacA4_2, tdcD, rocG_2, yhjQ, ftsP_1, yhhT, aatB_2, cysC, pcp, panE, hspA, alkA, mtnX, gshA, ykfA_3, dhaA_1, argP, hutG, lcfB_1, pdaC, ycaC_2, ftsI, gtaB, yhjR, bcsQ, yabJ_1, ybaB_2, tadA_1, gltP_1, lexA, ligD, ku_1, aacA4_3, doxA, yrbG, hoxH, asrB, ndhI, hypE, hypD, hypC, hypF, hypB, nixA, uspE_2, pacL, auaG, nqrF, cyaB_2, bepA, fabI_4, pafA_2, macA, fcl, pds, btuD_3, arnC_3, rfbC, garP, recB, dprE1, udk_3, qseC, gloB_2, emrA_2, oprM_5, yyaP_1, clcA_3, lacG, guaB, tadA_2, mscS, yyaP_2, ybaB_3, htrA, hpf_2, ydaD, tolB_2, derI, yveA, ibpA, glpE, ftsP_2, pah, hrp1_2, sthA, ynjF, yabJ_2, dadA, bioD1, bioH_1, cmk, thiK, fabH, dnaG_2, intA, dhaA_2, golD, bioH_2, pksC, xerC, sasA_10, gstB, gadC_4, gbpA, atm1, aaeB, korB, atoC_3, dauA, ampG_1, tam_3,*

		gdhA, alx_2, rlpA_3, malT, adc_2, ankX, arcB_3, lcfB_2, oprM_6, todT_3, phaB_5, ampG_2, spxA, gltP_2, kdpB_4, sasA_11, ku_2, rcsC_6, mnaT, csdA, vldW, carA_5, arnC_4, yceF, dps2_2, lcfB_3
		***L. anisa* genes not found in 9fVS26:** *catD_1, malT_1, ackA_1, xpkA_1, atpD_1, atpC_1, atpB_1, atpF_1, atpA_1, atpG_1, ftsP, cusA, cusB, lexA_1, cpbD_1, xerC_1, xerC_2, xerC_3, xerC_4, yhaJ_1, kmo, ygiD, algC_1, ldh_1, sufS_1, sspB, sspA_1, petC, petA, hemX, ybaB, rocG, fcl_1, guaB_1, yhaJ_2, panD_1, cysK, recF_1, osmY, atpB_2, atpF_2, atpH_3, atpA_2, atpG_2, atpD_2, atpC_2, glpQ, catD_2, cmpR, tagH, epsL, epsM, per, mhpC, mta, cyaB, traM_1, traL_1, traK_1, traJ_1, traI_1, traG_1, virB4_1, lexA_2, egtB_1, egtB_2, gshA_1, hemH_1, purK_1, purE_1, ndhB_1, gltR, apxIB_1, edd_1, glsA1_1, bioD1_1, phrA_1, crtK-2, phrB, ufaA1, purK_2, purE_2, ndhB_2, apxIB_2, panD_2, ddl, ygeA, mdrP, rizA, xpkA_2, aacA4, lon2, ephD, bcr, rimL, paaK, sufS_2, glaH, gshA_2, livK, rssB, csbC_1, kdnA, neoG, csbC_2, yveA_1, ohrA, adh, cda, cspV, dnaG, lolE_1, ldh_2, dps2, motA_1, recF_2, xerC_5, xerD_1, xerD_2, wecD_1, csrA_3, thiK_1, desA, baeB, baeC_1, novP, lolE_2, hin_1, lexA_3, aatB, cmk_1, yofA, glaR_1, fixL, cheB_3, ibpA_1, serA, xerD_3, xerD_4, xerC_6, gshA_3, rimJ, dhaA, srkA_3, ttuB, hutG_1, lcfB, htrA_1, ycaC, ftsI_1, gtaB_1, yabJ, mmcO, motA_2, xerC_7, xerD_5, xerD_6, xerC_8, xerD_7, xerD_8, ahpD_1, argT, bepA_1, pafA, pgl_1, spsI, fcl_2, dadA_1, mshA_3, rmlC, gudP, addA, dprE1_1, motB_3, tylM1, mltD, gloB, emrA, yyaP, fadA_3, add, araQ, guaB_2, tadA, gltC, hchA, cvfB, cspA_3, tolB, dltA_3, rpiB, yveA_2, ibpA_2, copB, copA_3, hutG_2, hrp1, thi4, slrP, dadA_2, yajO, bioD1_2, bioH, cmk_2, thiK_2, oprB, fabH_1, baeC_2, xerC_9, dgt, gstA, bioD1_3, glsA1_2, edd_2, besD, ccmH, dauA_1, ampG, sfp, csrA_4, dtpA_4, pal_3, dauA_2, phrA_2, ibpB, htrA_2, spkD, dprE1_2, malT_2, prs_3, cadA, adc, sspA_2, intS_3, csrA_5, celA, gtaB_2, algC_2, wbiB, bepA_2, galE1, ywqF, yfcJ, rayT, yfgD, gltP, ligD_1, ligD_2, ku, mshD_3, tycC, maf, lexA_4, mrgA, hin_2, dltA_4, fabG_6, mepM, spmB, spmA, pepA_1, holC, pepA_2, murJ, rpsT, uvrC, gacA, phhA, cinA, obg, rpmA, rplU, rplY, pth, ychF, ispB, cpbD_2, proP_6, esiB_3, traM_2, traL_2, traK_2, traJ_2, traI_2, traD, traG_2, virB4_2, csrA_6, xerC_10, ftsI_2, mecI, thrB, rhtC, hpf, smc_6, secA_3, ahpD_2, wecD_2, hemH_2, ppaX, csbC_3, edd_3, pgl_2, fabH_2, oleD, ahlD, ttgC, acpP_5, murU, ydfG, pepA_3, alx, frsA, glaR_2, tmoT, fosB, glsA, ackA_2, srfAB*
8cVS16^{T} *vs* *L. pneumophila*	1595 *vs* 1508	**8cVS16 genes not found in *L. pneumophila* SG1:** *murU_1, murU_2, chrA, ykfA_1, sdh, leuC, psuK, ybhF, ttgI_1, gpx1, hit, mdtK, pepA, alx_1, iucD, glaR, todT_1, yecN, per1, nat, budB, gabD_1, glsA1, ykfA_2, fabI_1, yacG, ytcD, ycdF, xylE, tolB_1, cbpD, rssB_1, divK, gatA_1, ypeA, oatA_1, fdhA, argE_1, yffB, aacA4_1, hss_1, rssB_2, chaB, yfiC, MENG, yhcG, desA3, kmo_1, aatB_1, gap, cheW, cheR1, cheB_1, cyaB_1, gpmA, nprV, rcsB, rcp1_1, rcp1_2, cph1, dauR, tdcB, resA_1, fadI, arcB_1, proC_1, bepG, atpC, atpD, atpG, atpA, atpF, atpB, yidD, resA_2, hap, yhdE, msrA, aroK_1, parA, ybgC_1, algA_1, rna, clcA_1, oatA_2, wbpI, capD, rmlD_1, wecA, hldD, wzxE, chrR, cspC, rocG_1, hinT, ybaB_1, cca_1, czcD_1, mhqR, petC_1, petC_2, amiC_1, yggR, dltA_1, ydgA, hrp1_1, ligd, cheB_2, cmdF, clcA_2, doeB, dltA_2, pknD_1, dgkA, rhtC_1, tcmN, kmo_2, nudC_1, tetA, czcD_2, slyA_1, nhaK, fmdA, adhT, dapL, cadI_1, nhaP, slyA_2, asnB, treA, gloB_1, pafA_1, rcsA, ctpF, uspE_1, nhaA, acr3, arsC, cadI_2, nfdA, ycaC_1, dnaG_1, mdh_1, mmpA, bioC, mshA_1, rspB, ribBA_1, pagN, dps2_1, adc_1, bcd, aacA4_2, phaB_4, fabI_2, fabI_3, tdcD, rocG_2, hmp_1, yhjQ, ftsP_1, intS_1, psdht, yhhT, aatB_2, cysC, yfeW_1, pcp, greB, sasA_4, rstA, rihA, panE, hdfR, nanT_1, phnY, phnZ, gamA, asnC, asnA, cidA, cynR, dgdA, cspA_1, sasA_5, todT_2, sasA_6, hspA, rhaS, alkA, pdxB_1, mtnX, ftsH4, dhpH, rutB, fbiB_1, ykfA_3, speE_3, inhA, arnC_1, guaD, tqsA, dhaA_1, dbpA_1, cysZ, pnoA, tdh, lcfB_1, pcpR, alr, dapE, dapD_1, ycaC_2, ftsI, pdxB_2, pmdD, kimA, fixB, aroK_2, proC_2, bcsG, bcsC, gtaB, yhjR, bcsQ, bcsA, bcsB, bcsZ, yabJ_1, thiS, moeZ, ybaB_2, tadA_1, intS_2, gltP_1, lexA, umuC, ligD, ku_1, aacA4_3, doxA, srpC, fbiB_2, kdpB_3, uspE_2, pacL, atl, auaG, nqrF, cyaB_2, sasA_7, artI, cmoA, thiM, menI, aes, nanT_2, norM, ribBA_2, fabI_4, pafA_2, acdA, bioF_1, yghO, pimA, ttgF, tagG, fcl, gmd_1, algA_2, rfbG, rfbF, pds, patA, gmd_2, arnC_2, mshA_2, arnC_3, rmlD_2, rffH, lysA_1, recB, lysA_2, mdh_2, flhB, flhA_1, lysS, cpoB_1, gloB_2, farB, emrA_1, yyaP_1, degQ, gabD_2, sufA, gatA_2, artP_1, clcA_3, ppsC, tadA_2, mscS, yyaP_2, fusA_1, appB, ttgI_2, ybaB_3, hpf_1, ybiT, cspA_2, pknD_2, ydaD, csrA2, sdhE, rpoE, ybgC_2, tolB_2, bamB_1, derI, yveA, dacB, adiC, ibpA, glpE, ftsP_2, yvgN, pah, hrp1_2, csd, yjjG, sthA, ynjF, COQ3_3, yabJ_2, menH_3, dadA, FCS1, amiC_2, rcsC_4, carA_3, ydjP, cpg2_1, speE_4, bioD1, bioH_1, bioF_2, pdeG, secA_1, dacC_1, ybeZ, sasA_8, lapB, fabG_3, fabH, lpxB, esiB, dnaG_2, intA, dhaA_2, nhaP2, golD, bioH_2, kynu, yybR, potE, erg, pksC, hisH, hisF, kup, xerC, dacC_2, secA_2, mtfA, sasA_9, mmpL3, hmp_2, menH_4, gbpA, rbn_1, alkB2, carA_4, cca_2, slyA_3, katA, atm1, yijE, hipA, aaeB, aaeA, hisC_3, rhaR, atoC_3, thiD, dauA, ampG_1, cpoB_2, gdhA, alx_2, rlpA_3, pbpE, dbpA_2, capB_3, rcsC_5, lgrD, malT, bamB_2, adc_2, artP_2, argF, ybaA, arcB_2, flp, arcB_3, argE_2, lcfB_2, lrp, todT_3, phaB_5, ampG_2, caiA, spxA, gltP_2, kdpA, kdpB_4, kdpC, kdpD, flhA_2, fabG_4, sasA_10, rcp1_3, ku_2, aldH1, rcsC_6, cpg2_2, selO, mnaT, sodC1, csdA, vldW, ahcY_3, carA_5, aroH, arnC_4, decR, iaaA, cphB, ybaL, yfeW_2, dipZ, madD, ndh, lysP, proP_5, dam, sauU_3, dps2_2, nanT_3, arnT, ctb, cydB, dapD_2, lcfB_3, dltC, fabG_5, nphT7, emrD_1, cpdA_3, aroK_3, hss_2, lpxA, rhtC_2, hpf_2, fpr, emrD_2, phaC_5, smc_5, ompR, sasA_11, mdtC_1, pgl, stp, emrA_2, oprM_4, sadH, lemA, mdtA_5, oprM_5, oprM_6, mdtC_2, rbn_2, pasI, lpxL, acpP_4, nudC_2, fusA_2, murU_3*

		**L. pneumophila SG1 genes not found in 8cVS16:** resA, hap_1, cat, merR, aroH_1, ddl_1, ydeN, msrA_1, ynjF_1, cdgJ, gapA, yedI, cynR_1, mhpA, pbuE, ddaF, rbsB, frcA, rbsC, dapE_1, yfdE, katG2, cydB_1, phrB, pknD, crtK-2, lrgA, cynR_2, purK, ndhB, gltC, lagD, yveA_1, mdlC, sidE, bpoC, glsA, yfgD, yhhQ, ttgC_1, emrA, yfbR, fosA, ydfG, murU, secE, fusA, lpxL_1, ddl_2, osmY, ogt, nagB, cspD, ttgC_2, cfiA, dmpP, hpf, artJ_3, argI, add, cdsA, lpxA_1, ytbE, fosB, plsC, spmA, spmB, mepM, pabC, ydjZ, arnT_1, nanT, dam_1, ybhF_1, norW, adc, artJ_4, ybhF_2, dps2, gsiA, tdh_1, cusS, czcR, alr-1, hisF_1, hisH_1, neuA, neuB_1, epsM, rmlD, rmlA, rfbB, tagO, neuB_2, kpsM, wbbL, blh, patA_1, ampG, dauA_1, rsbV, ybgI, lolC, ccmH, lapB_1, gltP, dapE_2, dapD, yihG, kmo, ycaC, ftsI_1, pdxB, etfA, aroK, csrA2_1, cadA_1, cadA_2, cusB_1, cusA_1, ftsP, hmp, atpG_1, atpA_1, atpF_1, atpB_1, atpC_1, atpD_1, esiB_1, intS, yhcG_1, xerC_1, murJ_1, yqaB, cusB_2, cusA_2, msrB_1, kaiB, kaiC, mltD_1, fliY, artJ_5, guaD_1, ousA, argE, cutD, esiB_2, zraR, bphD, ribBA, kup_1, hisF_2, hisH_2, cydB_2, proX, slyA, rhtC, yhcG_2, Int-Tn, yedK, lexA_1, umuC_1, vsr, recD2, virB9, virB4, csrA2_2, lexA_2, mltD_2, gloB, yejM, spnN, degP, gabD, esiB_3, gspH, lpxB_1, calB, ycdF_1, fabH_1, gudP, arnC, arnT_2, udp, lapB_2, cpxA, yciV, secA, zapD, bioF, bioH, bioD, decR_1, dacC, dat, apxIB, hepA, hlyD, yddE, dtpA_4, bamB, lapB_3, artJ_6, sthA_1, moeZ_1, tolB, ybgC, algU, yfiC_1, mta, ftsI_2, blaI, ahlD, oleD, fabH_2, mneA, thrC, galP, empA, dcsG, glgE, ung, umuC_2, lexA_3, cpg2, tadA, tdh_2, clcA, gatA, iscA, lysU, flhA, flhB_1, fliP, lysA, addA, acdA_1, gmhB, moeZ_2, pafA, fabI, xerC_2, clsB, lgoT, clcB, tgpA, pilE1_1, pilE1_2, hipA_1, yfiC_2, pksJ, tycC, acrC, mmgB, dltC_1, cypB, yxaF, yhbO, setA, guaD_2, yyaP, yabJ, proC, aroH_2, cpoB, xerC_3, atm1_1, atm1_2, dkgA, mazG, dam_2, traD, traN, traC, traV, csrA_3, lexA_4, msrA_2, msrB_2, ynjF_2, sthA_2, msrAB, dbpA, kup_2, czcA_3, czcC_3, pcpR_1, aadK, atm1_3, sdeA_1, sidJ_1, sdeA_2, ycgJ, rhaS_1, oqxB13, dltA, tam_4, alx, ibpA_1, ibpA_2, dauA_2, cynT, dtpA_5, patA_2, chiA, esiB_4, dltC_2, fabH_3, bcr_1, dhaT, addB, azoR1, tam_5, dagK, sodC, mdh, nudE, aam, dnaG, hipA_2, leuO, mgtA, dmlR_3, kup_3, yveA_2, katG1, aacA4, pcpR_2, fenF, bcr_2, elaA, rcp1, drrA, sidD, ibpB, kynB, hss, cqsS_1, sidJ_2, decR_2, glbN, gcoB, rhaS_2, lexA_5, acdA_2, flhB_2, vanX, dprA, pepA_1, holC, pepA_2, murJ_2, rpsT, uvrC, gacA, phhA, cinA, obg, rpmA, rplU, rplY, pth, ychF, ispB, cfiB, yhcR, ghrB, leuB, tam_6, petC, petA, egtC_3, aseR, cqsA, cqsS_2, czcD, cca, ybaB, yajO, APOA1BP, cspE, lubX, tpm, mdtL_3, nylA, ravA, ycdF_2, fadM, algA, soj, hcaC, mshA, msrA_3, lpxL_2, lpxL_3, lpxA_2, lpxD_3, lpxB_2, asnO, rbn, hap_2, atpC_2, atpD_2, atpG_2, atpA_2, atpF_2, atpH_3, atpB_2, alkB1
9fVS26 *vs* *L. pneumophila*	1596 *vs* 1508	**9fVS26 genes not found in L. pneumophila SG1:** murU_1, murU_2, chrA, ykfA_1, sdh, leuC, psuK, ybhF, ttgI_1, gpx1, hit, mdtK, pepA, alx_1, iucD, glaR, todT_1, yecN, per1, nat, budB, gabD_1, glsA1, ykfA_2, fabI_1, yacG, ytcD, ycdF, xylE, tolB_1, cbpD, rssB_1, divK, gatA_1, ypeA, oatA_1, fdhA, argE_1, yffB, aacA4_1, hss_1, rssB_2, chaB, yfiC, MENG, yhcG, desA3, kmo_1, aatB_1, gap, cheW, cheR1, cheB_1, cyaB_1, gpmA, nprV, rcsB, rcp1_1, rcp1_2, cph1, dauR, tdcB, resA_1, fadI, arcB_1, proC_1, bepG, atpC, atpD, atpG, atpA, atpF, atpB, yidD, resA_2, hap, yhdE, msrA, aroK_1, parA, ybgC_1, algA_1, rna, clcA_1, oatA_2, wbpI, capD, rmlD_1, wecA, hldD, wzxE, chrR, cspC, rocG_1, hinT, ybaB_1, cca_1, czcD_1, aroK_2, hss_2, lpxA, rhtC_1, hpf_1, fpr, emrD_1, ompR, sasA_4, mdtC_1, pgl, stp, emrA_1, sadH, lemA, oprM_4, mdtC_2, rbn_1, pasI, lpxL, nudC_1, fusA_1, murU_3, mhqR, petC_1, petC_2, amiC_1, yggR, dltA_1, ydgA, hrp1_1, ligd, cheB_2, cmdF, clcA_2, doeB, dltA_2, pknD_1, dgkA, rhtC_2, tcmN, kmo_2, nudC_2, tetA, czcD_2, slyA_1, nhaK, fmdA, adhT, dapL, cadI_1, nhaP, slyA_2, asnB, treA, gloB_1, pafA_1, rcsA, ctpF, uspE_1, nhaA, acr3, arsC, cadI_2, nfdA, ycaC_1, dnaG_1, mdh_1, mmpA, bioC, mshA_1, rspB, ribBA_1, pagN, dps2_1, adc_1, bcd, aacA4_2, phaB_4, fabI_2, fabI_3, tdcD, rocG_2, hmp_1, yhjQ, ftsP_1, intS_1, psdht, yhhT, aatB_2, cysC, yfeW_1, pcp, greB, sasA_5, rstA, rihA, panE, hdfR, nanT_1, phnY, phnZ, gamA, asnC, asnA, cidA, cynR, dgdA, cspA_1, sasA_6, todT_2, sasA_7, hspA, rhaS, alkA, pdxB_1, mtnX, ftsH4, dhpH, rutB, fbiB_1, ykfA_3, speE_3, inhA, arnC_1, guaD, tqsA, dhaA_1, dbpA_1, cysZ, pnoA, tdh, lcfB_1, pcpR, mdtA_5, alr, dapE, dapD_1, ycaC_2, ftsI, pdxB_2, pmdD, kimA, fixB, aroK_3, proC_2, bcsG, bcsC, gtaB, yhjR, bcsQ, bcsA, bcsB, bcsZ, yabJ_1, thiS, moeZ, ybaB_2, tadA_1, intS_2, gltP_1, lexA, umuC, ligD, ku_1, aacA4_3, doxA, srpC, fbiB_2, kdpB_3, uspE_2, pacL, atl, auaG, nqrF, cyaB_2, sasA_8, artI, cmoA, thiM, menI, aes, nanT_2, norM, ribBA_2, fabI_4, pafA_2, acdA, bioF_1, yghO, pimA, ttgF, tagG, fcl, gmd_1, algA_2, rfbG, rfbF, pds, patA, gmd_2, arnC_2, mshA_2, arnC_3, rmlD_2, rffH, lysA_1, recB, lysA_2, mdh_2, flhB, flhA_1, lysS, cpoB_1, gloB_2, farB, emrA_2, oprM_5, yyaP_1, degQ, gabD_2, sufA, gatA_2, artP_1, clcA_3, ppsC, tadA_2, mscS, yyaP_2, fusA_2, appB, ttgI_2, ybaB_3, hpf_2, ybiT, cspA_2, pknD_2, ydaD, csrA2, sdhE, rpoE, ybgC_2, tolB_2, bamB_1, derI, yveA, dacB, adiC, ibpA, glpE, ftsP_2, yvgN, pah, hrp1_2, csd, yjjG, sthA, ynjF, COQ3_3, yabJ_2, menH_3, dadA, FCS1, amiC_2, rcsC_4, carA_3, ydjP, cpg2_1, speE_4, bioD1, bioH_1, bioF_2, pdeG, secA_1, dacC_1, ybeZ, sasA_9, lapB, fabG_3, fabH, lpxB, dnaG_2, intA, dhaA_2, nhaP2, golD, bioH_2, kynu, yybR, potE, erg, pksC, hisH, hisF, kup, xerC, dacC_2, secA_2, mtfA, sasA_10, mmpL3, hmp_2, menH_4, gbpA, rbn_2, alkB2, carA_4, cca_2, slyA_3, katA, atm1, yijE, hipA, aaeB, aaeA, hisC_3, rhaR, atoC_3, thiD, smc_5, dauA, ampG_1, cpoB_2, gdhA, alx_2, rlpA_3, pbpE, dbpA_2, capB_3, rcsC_5, lgrD, malT, bamB_2, adc_2, artP_2, argF, ybaA, arcB_2, flp, arcB_3, argE_2, lcfB_2, lrp, oprM_6, todT_3, phaB_5, ampG_2, caiA, spxA, phaC_5, gltP_2, kdpA, kdpB_4, kdpC, kdpD, flhA_2, fabG_4, sasA_11, rcp1_3, ku_2, aldH1, rcsC_6, cpg2_2, selO, mnaT, sodC1, csdA, vldW, ahcY_3, carA_5, aroH, arnC_4, decR, iaaA, cphB, ybaL, yfeW_2, dipZ, madD, ndh, lysP, proP_5,

		dam, sauU_3, dps2_2, nanT_3, arnT, ctb, cydB, dapD_2, lcfB_3, dltC, fabG_5, nphT7, acpP_4, emrD_2, cpdA_3
		***L. pneumophila* SG1 genes not found in 9fVS26:** *resA, hap_1, cat, merR, aroH_1, ddl_1, ydeN, msrA_1, ynjF_1, cdgJ, gapA, yedI, cynR_1, mhpA, pbuE, ddaF, rbsB, frcA, rbsC, dapE_1, yfdE, katG2, cydB_1, phrB, pknD, crtK-2, lrgA, cynR_2, purK, ndhB, gltC, lagD, yveA_1, mdlC, sidE, bpoC, glsA, yfgD, yhhQ, ttgC_1, emrA, yfbR, fosA, ydfG, murU, secE, fusA, lpxL_1, ddl_2, osmY, ogt, nagB, cspD, ttgC_2, cfiA, dmpP, hpf, artJ_3, argI, add, cdsA, lpxA_1, ytbE, fosB, plsC, spmA, spmB, mepM, pabC, ydjZ, arnT_1, nanT, dam_1, ybhF_1, norW, adc, artJ_4, ybhF_2, dps2, gsiA, tdh_1, cusS, czcR, alr-1, hisF_1, hisH_1, neuA, neuB_1, epsM, rmlD, rmlA, rfbB, tagO, neuB_2, kpsM, wbbL, blh, patA_1, ampG, dauA_1, rsbV, ybgI, lolC, ccmH, lapB_1, gltP, dapE_2, dapD, yihG, kmo, ycaC, ftsI_1, pdxB, etfA, aroK, csrA2_1, cadA_1, cadA_2, cusB_1, cusA_1, ftsP, hmp, atpG_1, atpA_1, atpF_1, atpB_1, atpC_1, atpD_1, intS, yhcG_1, xerC_1, murJ_1, yqaB, cusB_2, cusA_2, msrB_1, kaiB, kaiC, mltD_1, fliY, artJ_5, guaD_1, ousA, argE, cutD, zraR, bphD, ribBA, kup_1, hisF_2, hisH_2, cydB_2, proX, slyA, rhtC, yhcG_2, Int-Tn, yedK, lexA_1, umuC_1, vsr, recD2, virB9, virB4, csrA2_2, lexA_2, mltD_2, gloB, yejM, spnN, degP, gabD, esiB_3, gspH, lpxB_1, calB, ycdF_1, fabH_1, gudP, arnC, arnT_2, udp, lapB_2, cpxA, yciV, secA, zapD, bioF, bioH, bioD, decR_1, dacC, dat, apxIB, hepA, hlyD, yddE, dtpA_4, bamB, lapB_3, artJ_6, sthA_1, moeZ_1, tolB, ybgC, algU, yfiC_1, mta, ftsI_2, blaI, ahlD, oleD, fabH_2, mneA, thrC, galP, empA, dcsG, glgE, ung, umuC_2, lexA_3, cpg2, tadA, tdh_2, clcA, gatA, iscA, lysU, flhA, flhB_1, fliP, lysA, addA, acdA_1, gmhB, moeZ_2, pafA, fabI, xerC_2, clsB, lgoT, clcB, tgpA, pilE1_1, pilE1_2, hipA_1, yfiC_2, pksJ, tycC, acrC, mmgB, dltC_1, cypB, yxaF, yhbO, setA, guaD_2, yyaP, yabJ, proC, aroH_2, cpoB, xerC_3, atm1_1, atm1_2, dkgA, mazG, dam_2, traD, traN, traC, traV, csrA_3, lexA_4, msrA_2, msrB_2, ynjF_2, sthA_2, msrAB, dbpA, kup_2, czcA_3, czcC_3, pcpR_1, aadK, atm1_3, sdeA_1, sidJ_1, sdeA_2, ycgJ, rhaS_1, oqxB13, dltA, tam_4, alx, ibpA_1, ibpA_2, dauA_2, cynT, dtpA_5, patA_2, chiA, esiB_4, dltC_2, fabH_3, bcr_1, dhaT, addB, azoR1, tam_5, dagK, sodC, mdh, nudE, aam, dnaG, hipA_2, leuO, mgtA, dmlR_3, kup_3, yveA_2, katG1, aacA4, pcpR_2, fenF, bcr_2, elaA, rcp1, drrA, sidD, ibpB, kynB, hss, cqsS_1, sidJ_2, decR_2, glbN, gcoB, rhaS_2, lexA_5, acdA_2, flhB_2, vanX, dprA, pepA_1, holC, pepA_2, murJ_2, rpsT, uvrC, gacA, phhA, cinA, obg, rpmA, rplU, rplY, pth, ychF, ispB, cfiB, yhcR, ghrB, leuB, tam_6, petC, petA, egtC_3, aseR, cqsA, cqsS_2, czcD, cca, ybaB, yajO, APOA1BP, cspE, lubX, tpm, mdtL_3, nylA, ravA, ycdF_2, fadM, algA, soj, hcaC, mshA, msrA_3, lpxL_2, lpxL_3, lpxA_2, lpxD_3, lpxB_2, asnO, rbn, hap_2, atpC_2, atpD_2, atpG_2, atpA_2, atpF_2, atpH_3, atpB_2, alkB1*
L. anisa<br>*vs*<br>*L. pneumophila*	1677<br>*vs*<br>1508	***L. anisa* genes not found in *L. pneumophila* SG1:** *catD_1, malT_1, fabI_1, ackA_1, xpkA_1, hmp_1, cusA, cusB, cpbD_1, xerC_4, slyA_1, czcD_1, yhaJ_1, nudC_1, tcmN, ygiD, dgkA, pknD_1, dltA_1, cmdF, algC_1, ldh_1, sufS_1, dltA_2, yggR, amiC_1, sspB, sspA_1, slyA_2, hemX, czcD_2, cca_1, hinT, rocG, cheB_1, cspA_1, chrR, wzxE, fcl_1, wecA, rmlD_1, capD, wbpI, oatA_1, clcA_1, guaB_1, rna, algA_1, ybgC_1, parA, yhaJ_2, msrA, yhdE, panD_1, cysK, hap, resA_1, recF_1, yidD, bepG, proC_1, arcB_1, glpQ, fadI, catD_2, cmpR, mshA_1, tagH, epsL, per, mhpC, pknD_2, resA_2, tdcB, rcp1_1, rcp1_2, gpmA, cyaB, cheB_2, gap, traM_1, traL_1, traK_1, traJ_1, traI_1, traG_1, virB4_1, egtB_1, egtB_2, gshA_1, hemH_1, purK_1, purE_1, ndhB_1, gltR, apxIB_1, edd_1, glsA1_1, bioD1_1, phrA_1, appB, ufaA1, purK_2, purE_2, ndhB_2, hdfR, apxIB_2, menH_3, panD_2, ddl, ygeA, mdrP, rizA, xpkA_2, MENG, yfiC, chaB, hss_1, yffB, lon2, ephD, argE_1, fdhA, bcr, rimL, oatA_2, paaK, sufS_2, glaH, gshA_2, gatA_1, livK, divK, rssB, csbC_1, kdnA, neoG, csbC_2, xylE, ohrA, fmdA, adh, dapL, cda, srpC, desA3, cspV, mdh_1, rcp1_3, mmpA, lolE_1, ldh_2, mshA_2, rspB, ribBA_1, motA_1, bcd, recF_2, xerC_5, xerD_1, xerD_2, wecD_1, thiK_1, desA, baeB, baeC_1, novP, lolE_2, intS_1, hin_1, intS_2, aatB, cmk_1, yfeW_1, greB, rstA, rihA, yofA, nanT_1, phnY, phnZ, gamA, asnC, asnA, dgdA, cynR, cidA, glaR_1, cspA_2, fixL, cheB_3, todT_1, rhaS, serA, xerD_3, xerD_4, xerC_6, pdxB_1, ftsH4, dhpH, gshA_3, rutB, fbiB_1, rimJ, inhA, arnC_1, guaD, tqsA, dhaA, dbpA_1, cysZ, pnoA, srkA_3, ttuB, tdh, hutG_1, lcfB, pcpR, alr, dapE, dapD_1, htrA_1, pdxB_2, pmdD, kimA, fixB, aroK_1, proC_2, bcsG, bcsC, gtaB_1, bcsA, bcsB, bcsZ, thiS, moeZ, mmcO, motA_2, xerC_7, xerD_5, xerD_6, xerC_8, xerD_7, xerD_8, psdht, fbiB_2, atl, ahpD_1, adiC, argT, cmoA, thiM, menI, aes, nanT_2, norM, bepA_1, ribBA_2, fabI_2, pgl_1, acdA, spsI, bioF_1, yghO, pimA, ttgF, aaeA, tagG, fcl_2, gmd_1, algA_2, rfbG, rfbF, dadA_1, patA, gmd_2, mshA_3, rmlC, rmlD_2, rffH, lysA_1, lysA_2, mdh_2, dprE1_1, flhB, flhA_1, motB_3, lysS, tylM1, cpoB_1, sasA_4, mltD, farB, degQ, gabD_1, fadA_3, sufA, gatA_2, artP_1, clcA_2, ppsC, araQ, guaB_2, fusA_1, ttgI_1, hchA, cvfB, ybiT, cspA_3, csrA2, sdhE, rpoE, ybgC_2, bamB_1, dltA_3, rpiB, dacB, copB, copA_3, yvgN, fabG_3, hutG_2, hrp1, csd, yjjG, COQ3_3, thi4, slrP, dadA_2, FCS1, amiC_2, ydjP, cpg2_1, speE_3, bioD1_2, bioF_2, pdeG, secA_1, dacC_1, ybeZ, sasA_5, lapB, cmk_2, thiK_2, oprB, fabG_4, lpxB, nhaP2, kynu, yybR, potE, erg, baeC_2, hisH, hisF, kup, xerC_9, dacC_2, dgt, secA_2, mtfA, sasA_6, mmpL3, gstA, hmp_2, menH_4, artI, rbn_1, alkB2, bioD1_3, glsA1_2, edd_2, cca_2, slyA_3, besD, katA, yijE, hipA, hisC_3, rhaR, thiD, smc_5, sfp, csrA_4, cpoB_2, pal_3, dbpA_2, capB_3, carA_3, phrA_2, htrA_2, pbpE, spkD, rcsC_4, lgrD, dprE1_2, malT_2, prs_3, cadA, bamB_2, artP_2, argF, sspA_2, ybaA, flp, arcB_2, argE_2, intS_3, sasA_7, csrA_5, celA, gtaB_2, algC_2, wbiB, bepA_2, galE1, ywqF, yfcJ, lrp, todT_2, phaB_4, caiA, rayT, aroK_2, kdpA, kdpB_3, kdpC, kdpD, flhA_2, fabG_5, ligD_1, ligD_2, ku, aldH1, rcsC_5, cpg2_2, selO, mshD_3, sodC1, ahcY_3, carA_4, tetA, aroH, arnC_2, maf, decR, iaaA, cphB, ybaL, yfeW_2, dipZ, madD, ndh, umuC, lysP, proP_5, dam, sauU_3,*

	mrgA, nanT_3, arnT, ctb, cydB, hin_2, dapD_2, dltA_4, dltC, fabG_6, nphT7, acpP_4, emrD_1, murJ, cpbD_2, proP_6, traM_2, traL_2, traK_2, traJ_2, traI_2, traG_2, virB4_2, csrA_6, xerC_10, mecI, thrB, aroK_3, hss_2, lpxA, fpr, emrD_2, phaC_5, smc_6, secA_3, ahpD_2, ompR, sasA_8, wecD_2, hemH_2, mdtC_1, ppaX, csbC_3, edd_3, pgl_2, stp, mdtA_5, oprM_4, ttgC, mdtC_2, rbn_2, pasI, lpxL, acpP_5, nudC_2, fusA_2, chrA, ykfA_1, leuC, ybhF, ttgI_2, gpx1, hit, pepA_3, frsA, iucD, glaR_2, tmoT, sasA_9, yecN, per1, speE_4, budB, gabD_2, ykfA_2, ackA_2, fabI_3, yacG, cpdA_3, nhaK, srfAB
	**L. pneumophila SG1 genes not found in L. anisa**: recF, resA, hap_1, cat, yhaJ, merR, aroH_1, ddl_1, ydeN, msrA_1, ynjF_1, cdgJ, gapA, yedI, cynR_1, mhpA, pbuE, ddaF, rbsB, frcA, rbsC, dapE_1, yfdE, katG2, cydB_1, pknD, phrA, lrgA, cynR_2, purK, purE, ndhB, lagD, mdlC, sidE, bpoC, yhhQ, ttgC_1, yfbR, fosA, secE, fusA, lpxL_1, gshA, ddl_2, ogt, nagB, edd, csbC, hemH, cspD, ttgC_2, cfiA, dmpP, artJ_3, argI, cdsA, lpxA_1, ytbE, plsC, pabC, ydjZ, sufS, arnT_1, nanT, dam_1, ybhF_1, ybhR_1, norW, artJ_4, ybhF_2, ybhR_2, gsiA, ankX, tdh_1, hutG, pdaC, cusS, czcR, alr-1, hisF_1, hisH_1, neuA, neuB_1, rfbC, rmlD, rmlA, rfbB, tagO, neuB_2, kpsM, wbbL, blh, patA_1, rsbV, ybgI, lolC, macA, lapB_1, dapE_2, dapD, yihG, yrbG, htrA, pdxB, etfA, aroK, korB, csrA2_1, cadA_1, cadA_2, cusB_1, cusA_1, hmp, intS, yhcG_1, murJ_1, tam_3, yqaB, cusB_2, cusA_2, msrB_1, kaiB, kaiC, mltD_1, fliY, artJ_5, guaD_1, potD, potB, potA, ousA, argE, cutD, zraR, bphD, bepA, ribBA, kup_1, hisF_2, hisH_2, cydB_2, proX, slyA, yhcG_2, Int-Tn, yedK, umuC_1, vsr, recD2, virB9, virB4, csrA2_2, qseC, mltD_2, yejM, spnN, degP, gabD, gspH, lpxB_1, calB, ycdF_1, arnC, arnT_2, thiK, udp, cmk, lapB_2, cpxA, yciV, secA, zapD, bioF, bioD, decR_1, dacC, dat, apxIB, hepA, hlyD, yddE, bamB, lapB_3, artJ_6, sthA_1, moeZ_1, ybgC, algU, yfiC_1, blaI, mneA, thrC, galP, empA, dcsG, glgE, ung, umuC_2, cpg2, guaB, lacG, tdh_2, clcA, gatA, iscA, lysU, flhA, flhB_1, fliP, dprE1, argP, lysA, garP, acdA_1, gmhB, moeZ_2, udk_3, fabI, clsB, lgoT, clcB, tgpA, gstB, pilE1_1, pilE1_2, gadC_4, hipA_1, yfiC_2, pksJ, acrC, mmgB, dltC_1, cypB, yxaF, yhbO, setA, guaD_2, proC, aroH_2, yceF, cpoB, atm1_1, atm1_2, dkgA, mazG, dam_2, traN, traC, traV, msrA_2, msrB_2, ynjF_2, sthA_2, msrAB, dbpA, kup_2, czcA_3, czcC_3, pcpR_1, aadK, atm1_3, sdeA_1, sidJ_1, sdeA_2, ycgJ, rhaS_1, oqxB13, dltA, tam_4, cynT, dtpA_5, patA_2, chiA, esiB_4, dltC_2, fabH_3, bcr_1, dhaT, ackA, addB, ldh, lolE, azoR1, motA, tam_5, dagK, sodC, ahpD, mdh, nudE, aam, hipA_2, leuO, mgtA, dmlR_3, kup_3, katG1, pcpR_2, fenF, bcr_2, elaA, rcp1, drrA, sidD, hoxH, asrB, ndhI, hypE, hypD, hypC, hypF, hypB, nixA, algC, kynB, hss, cqsS_1, sidJ_2, decR_2, aspC, glbN, gcoB, rhaS_2, lexA_5, hin, acdA_2, flhB_2, vanX, dprA, murJ_2, cfiB, yhcR, ghrB, leuB, tam_6, sspA, egtC_3, aseR, cqsA, cqsS_2, czcD, cca, APOA1BP, cspE, lubX, tpm, mdtL_3, guaC, nylA, ravA, ycdF_2, fadM, algA, soj, hcaC, mshA, msrA_3, panD, lpxL_2, lpxL_3, lpxA_2, lpxD_3, lpxB_2, btuD_3, asnO, cbs, rbn, hap_2, alkB1

Table S5. List of virulence genes shared among the *Legionella* species belonging to the same clade: 8cVS16^{T}, 9fVS26, *Legionella anisa* WA-316-C3 ATCC35292^{T}, *Legionella bozemanae* WIGA ATCC 33217^{T}, *Legionella parisiensis* PF-209C-C2 ATCC35299^{T}, *Legionella tusconensis* 1087AZH ATCC49180^{T}, *Legionella wadsworthii* 81-716A ATCC 33877^{T}. All species show blue-white autofluorescence.

**Present genes***	htpB, pilB, pilC, pilD, pilE, pilM, pilN, pilO, pilP, pilQ, mip, enhA, ccmA, ccmC, ccmD, ccmE, ccmF, feoB, frgA, iraA, iraB, lbtB, rpoS, csrA, letS, relA, dotA, dotB, dotC, dotD, icmB/dotO, icmC/dotE, icmD/dotP, icmE/dotG, icmF, icmG/dotF, icmH/dotU, icmJ/dotN, icmK/dotH, icmL/dotI, icmN/dotK, icmO/dotL, icmP/dotM, icmQ, icmS, icmV, icmW, icmX, lvgA, lspC, lspD, lspE, lspF, lspG, lspH, lsp
**Missed genes**	lidL, drrA/sidM, laiE, lepA, lidA, ralF, sdbA, sdbB-like, sdbB, sdbC, sdcA, sdeA/laiA, sdeB/laiB, sdeC/laiC, sdeD/laiF, sdhA, lvhB7, lvhB5, lvhB3, icmR, lbtA, sidA, sidB, sicC, sidD, sidE-like, sidE/laiD, sidF, sidG, sidH, vipA, vipD1, vipD2, vipD3, vipE, wipB, wipC, ylfB, rtxA

* *wcbB* gene is present only in 8cVS16^{T} and 9fVS26 isolates.
